# Aging represses oncogenic KRAS-driven lung tumorigenesis and alters tumor suppression

**DOI:** 10.1038/s43587-025-00986-z

**Published:** 2025-11-04

**Authors:** Emily G. Shuldiner, Saswati Karmakar, Min K. Tsai, Jess D. Hebert, Yuning J. Tang, Laura Andrejka, Maggie R. Robertson, Minwei Wang, Colin R. Detrick, Hongchen Cai, Rui Tang, Christian A. Kunder, David M. Feldser, Dmitri A. Petrov, Monte M. Winslow

**Affiliations:** 1 Department of Biology, Stanford University, Stanford, CA USA; 2 Department of Genetics, Stanford University School of Medicine, Stanford, CA USA; 3 Cancer Biology Program, Stanford University School of Medicine, Stanford, CA USA; 4 Department of Cancer Biology, Perelman School of Medicine, University of Pennsylvania, Philadelphia, PA USA; 5 Cell and Molecular Biology Graduate Group, Perelman School of Medicine, University of Pennsylvania, Philadelphia, PA USA; 6 Abramson Family Cancer Research Institute, Perelman School of Medicine, University of Pennsylvania, Philadelphia, PA USA; 7 Department of Pathology, Stanford University School of Medicine, Stanford, CA USA; 8 The Chan Zuckerberg BioHub, San Francisco, CA USA

**Keywords:** Ageing, Cancer models, Tumour-suppressor proteins

## Abstract

Most cancers are diagnosed in people over 60 years of age, but little is known about how age impacts tumorigenesis. While aging is accompanied by mutation accumulation (widely understood to contribute to cancer risk) it is associated with numerous other cellular and molecular changes likely to impact tumorigenesis. Moreover, cancer incidence decreases in the oldest part of the population, suggesting that very old age may reduce carcinogenesis. Here we show that aging represses oncogenic KRAS-driven tumor initiation and growth in genetically engineered mouse models of human lung cancer. Moreover, aging dampens the impact of inactivating many tumor suppressor genes with the impact of inactivating PTEN, a negative regulator of the PI3K–AKT pathway, weakened disproportionately. Single-cell transcriptomic analysis revealed that neoplastic cells in aged mice retain age-related transcriptomic changes, showing that the impact of age persists through oncogenic transformation. Furthermore, the consequences of PTEN inactivation were strikingly age-dependent, with PTEN deficiency reducing signatures of aging in cancer cells and the tumor microenvironment. Our findings underscore the interconnectedness of the pathways involved in aging and tumorigenesis and document tumor-suppressive effects of aging that may contribute to the deceleration in cancer incidence with age.

## Abstract (web summary)

The mechanisms that impact tumorigenesis during aging are incompletely understood. Here Shuldiner et al. show that in mice, aging represses KRAS-driven lung tumorigenesis and dampens the impact of inactivating many tumor suppressor genes, which may contribute to the deceleration in cancer incidence with extreme age in humans.

## Main

Aging and cancer are complex, interrelated processes^1–3^. Epidemiologically, cancer incidence increases with age but does so non-monotonically, with incidence rates rising exponentially before leveling off and ultimately declining in the oldest part of the population^4–8^. While the age-related increase has long been attributed to the progressive accumulation of driver mutations^9,10^, the deceleration in cancer incidence and mortality among the oldest remains a puzzle and subject of debate^11–13^. Moreover, cancer and aging share many molecular hallmarks, suggesting that there may be additional mechanistic links between these processes^14,15^. Disentangling the complex interplay between aging and tumorigenesis remains a key conceptual and practical challenge for the field.

Lung cancer is the leading cause of cancer death and is highly age-dependent^7^. Autochthonous tumors in genetically engineered mouse models of lung cancer grow within their natural setting and recapitulate critical features of human tumors^16,17^. These tractable systems are uniquely suited to investigate the effects of aging on tumorigenesis because they allow for the controlled induction of cancer-driver mutations. These models can thus largely separate the age-related increase in the probability of driver mutations from other potential age-related effects on tumorigenesis and eliminate many of the confounding factors that complicate inference from epidemiological and clinical data.

Here we integrate autochthonous models of human lung cancer with tumor barcoding, somatic CRISPR/Cas9 genome editing^18,19^, and single-cell transcriptomic analyses to precisely quantify the effects of aging on tumor initiation, subsequent tumor growth and the functional landscape of tumor suppression. We find that aging represses lung tumorigenesis and lessens the impact of altering several tumor-suppressive pathways. Molecular analyses show that the effects of inactivation of PTEN on both cancer cells and the tumor microenvironment are strongly impacted by age. Our work suggests that aging profoundly shapes tumor biology and is consistent with a model in which cancer incidence patterns reflect the competing effects of protumor driver mutation accumulation and antitumor effects of tissue aging.

## Results

### Aging represses oncogenic KRAS-driven lung tumor initiation and growth

**Fig. 1:**
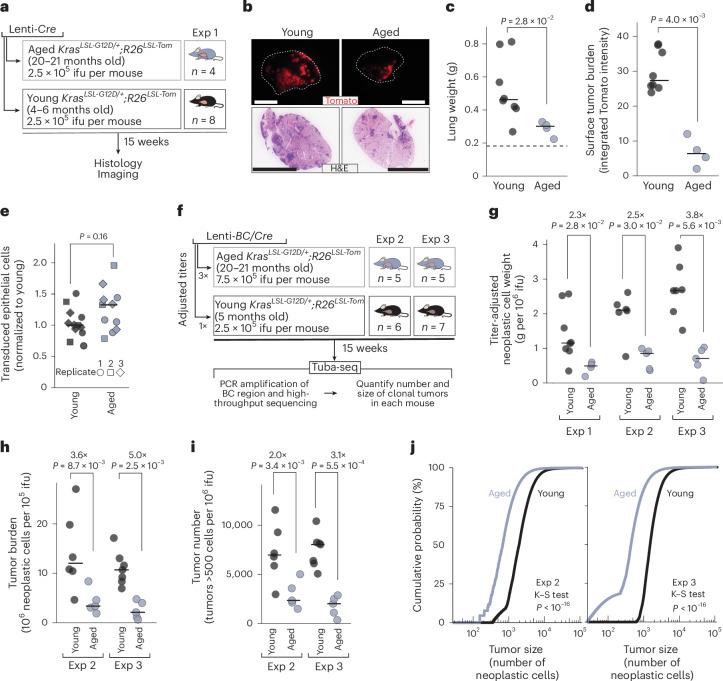
Age reduces oncogenic KRAS-driven lung tumor initiation and growth. **a**, Initiation of KRAS-driven lung tumors in young and aged *Kras*^*LSL-G12D/+*^*;R26*^*LSL-Tomato*^ mice with Lenti-*Cre* (Experiment (Exp) 1). Age and number of mice are indicated. See Supplementary Fig. 1a for timeline of mouse aging and tumor initiation and Supplementary Fig. 1b for exact ages and sexes. ifu, infectious units. **b**, Representative Tomato fluorescence and hematoxylin and eosin (H&E) images of lungs from young and aged mice. Scale bars, 5 mm. Dashed lines outline tissue. **c**, Tumor-bearing lung weights of young (*n* = 8) and aged (*n* = 4) mice. Each dot is a mouse and the bars indicate the median values. Normal lung weight is indicated by the dashed line. **d**, Fluorescence-based quantification of tumor burden in young (*n* = 8) and aged (*n* = 4) mice. Each dot is a mouse and bars indicate median values. *P* value obtained using a two-sided Wilcoxon rank-sum test. **e**, Quantification of transduction efficiency in young (*n* = 12) and aged (*n* = 11) mice merged across 3 replicate experiments. Values are normalized to the median percentage of transduced epithelial cells per experiment. Each point is a mouse; the shapes indicate the replicate experiment. *P* value obtained using a two-sided Wilcoxon rank-sum test. **f**, Initiation of KRAS-driven lung tumors in young and aged *Kras*^*LSL-G12D/+*^*;R26*^*LSL-Tomato*^ mice using threefold higher titers of Lenti-*BC/Cre* in the aged mice. Two replicate experiments were performed (Experiments 2 and 3). Age and number of mice are indicated. See Supplementary Fig. 1d for exact ages and sexes. **g**, Estimated neoplastic cell weights of young (*n* = 8, 6 and 7) and aged (*n* = 4, 5 and 5) mice, normalized to the viral titer delivered. Neoplastic cell weights are tumor-bearing lung weights minus normal lung weight (median value from Supplementary Fig. 1c). Each dot is a mouse and the bars indicate median values. *P* values obtained using a two-sided Wilcoxon rank-sum test. **h**,**i**, Tumor burden (total number of neoplastic cells in clonal expansions >500 cells; **h**) and number of tumors (clonal expansions >500 cells; **i**) in young (*n* = 6 and 7) and aged (*n* = 5 and 5) mice in Experiments 2 and 3, quantified by Tuba-seq and adjusted for titer. *P* values obtained using a two-sided Wilcoxon rank-sum test. **j**, Empirical cumulative distribution functions of tumor sizes in young (*n* = 6 and 7) and aged (*n* = 5 and 5) mice. To account for the threefold higher titer delivered to the aged mice, this comparison includes the 10,000 largest tumors in each young sample and the 30,000 largest tumors from each aged sample. Tumors in aged mice are smaller than tumors in young mice in both experiments. K–S, two-sided asymptotic Kolmogorov–Smirnov test comparing the distribution of tumor sizes in young and aged mice. Source Data

To assess the impact of aging on lung tumorigenesis, we initiated tumors by intratracheal delivery of a lentiviral vector encoding Cre recombinase in young (4–6 months old) and aged (20–21 months old) mice with *Kras*^*LSL-G12D/+*^ and *Rosa26*^*LSL-Tomato*^ alleles^20,21^ (Fig. 1a and Supplementary Fig. 1a,b). The ages of the young and aged mice were chosen to correspond to early adulthood and the age at which most molecular phenotypes of aging emerge, respectively^22,23^. Fifteen weeks after tumor initiation, aged mice had a roughly threefold lower tumor burden as measured by lung weight and fluorescence imaging (two-sided Wilcoxon rank-sum tests, *P* = 0.028 and 0.0040; Fig. 1b–d). There was no difference in lung weight between age-matched non-transduced young and aged controls, confirming that weight-based metrics of tumor burden are not confounded by changes with age unrelated to tumor growth (two-sided Wilcoxon rank-sum test, *P* = 0.49; Supplementary Fig. 1c) To determine whether reduced efficiency of lentiviral transduction of aged lung epithelial cells could explain these results, we transduced young and aged wild-type mice with a Lenti-*GFP* vector followed by flow cytometry- and immunohistochemistry-based quantification of the percentage of transduced epithelial cells (Supplementary Fig. 2a). Transduction efficiency was similar in young and aged mice, demonstrating that the reduction in tumorigenesis with age is not driven by reduced lung epithelial cell transduction (Fig. 1e and Supplementary Fig. 2b,c).

To confirm that age reduces lung tumorigenesis and to generate samples for tumor barcoding coupled with high-throughput barcode sequencing (Tuba-seq)^18,19^, we initiated tumors with a barcoded Lenti-*Cre* vector (Lenti-*BC*/*Cre*) in two additional groups of young and aged mice (Fig. 1f and Supplementary Fig. 1d). We initiated lung tumors in aged mice with a threefold higher titer of Lenti-*BC*/*Cre* to match the tumor burden in young and aged mice and generate material for subsequent molecular analyses (Fig. 1f). As a result, these aged mice had lung weights similar to their young counterparts (Supplementary Fig. 1e,f). The magnitude of reduction in tumorigenesis in aged mice was consistent across all three experiments, with aged mice having a two- to threefold reduction in tumor burden, when accounting for differences in viral titer (two-sided Wilcoxon rank-sum test, *P* = 2.8 × 10^−2^, 3.0 × 10^−2^ and 5.6 × 10^−3^; Fig. 1g). Tumors from young and aged mice had similar histological grades^24^ (Supplementary Fig. 1g,h).

To precisely quantify the number of tumors and size of each clonal tumor in each mouse and thereby disentangle the impacts of aging on lung tumor initiation and growth, we extracted DNA from tumor-bearing lungs, amplified the barcode region from the integrated lentiviral vectors and high-throughput sequenced the amplicon. The number of tumors (number of unique barcoded growths) and the tumor burden (total number of neoplastic cells summed across all tumors) as quantified by Tuba-seq were consistently reduced in aged mice. Tumor number was reduced two- to threefold and tumor burden was reduced four- to fivefold (two-sided Wilcoxon rank-sum test *P* = 3.4 × 10^−3^, 5.5 × 10^−4^ for tumor number and *P* = 8.7 × 10^−3^, 2.5 × 10^−3^ for tumor burden; Fig. 1h,i). Notably, tumors were also significantly smaller in aged mice, suggesting that aging represses both lung tumor initiation and subsequent tumor growth (Kolmogorov–Smirnov (K–S) test *P* < 10^−16^; Fig. 1j). Tumor burden, number and size were reduced with age in both males and females, indicating that age represses tumorigenesis irrespective of sex (Supplementary Fig. 3a–f).

### Impacts of tumor suppressor gene inactivation change with age

**Fig. 2:**
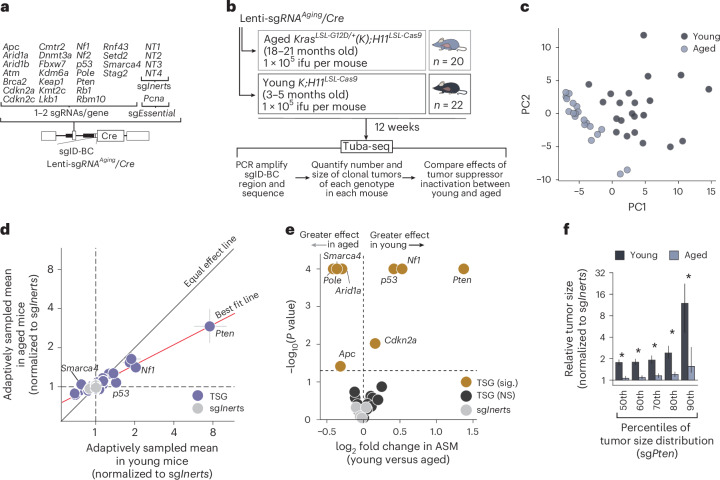
Age alters the landscape of tumor suppression. **a**, Barcoded Lenti-sg*RNA/Cre* vectors in the Lenti-sg*RNA*^*Aging*^*/Cre* pool included 1–2 vectors targeting each putative tumor suppressor, four vectors encoding inert (non-targeting) sgRNAs, and a vector with an sgRNA targeting an essential gene. **b**, Initiation of lung tumors in cohorts of young and aged *K;H11*^*LSL-Cas9*^ mice with Lenti-sg*RNA*^*Aging*^*/Cre*. Ages, genotype, lentiviral titer and mouse number are indicated. Mice were analyzed after 12 weeks of tumor growth. See Extended Data Fig. 1a for exact ages and sexes. **c**, Principal-component analysis of tumor-suppressive effects calculated within each mouse reveals separation of young and aged mice. Each dot is a mouse. **d**, ASM size of each tumor genotype normalized to the ASM of sg*Inert* tumors in young (*x* axis) versus aged (*y* axis) *K;H11*^*LSL-Cas9*^ mice transduced with Lenti-sg*RNA*^*Aging*^*/Cre*. ASM is a summary metric of tumor fitness that integrates the impact of inactivating each gene on tumor size and number (Methods). Error bars indicate 95% confidence intervals around the point estimate of the test statistic (center of bars). Confidence intervals were calculated by nested bootstrapped resampling across 22 young and 20 aged mice. ‘Equal effect line’, *y* = *x*. ‘Best fit line’, regression of aged ASM onto young ASM for all tumor suppressor genes (TSGs). **e**, Volcano plot showing fold change in the ASM of tumors of each genotype in the young versus aged *K;H11*^*LSL-Cas9*^ cohorts. Reported ASMs for each genotype are normalized to the ASM of sg*Inert* tumors. TSGs with a significantly different impact in young and aged mice (two-sided false discovery rate (FDR)-adjusted *P* < 0.05) are highlighted in gold and labeled. sg*Inert* vectors are highlighted in light gray. *P* values were calculated using nested bootstrap resampling across 22 young and 20 aged mice. **f**, Adaptively sampled sizes of tumors initiated with sg*Pten* vectors in young and aged *K;H11*^*LSL-Cas9*^ mice transduced with the Lenti-sg*RNA*^*Aging*^*/Cre* pool at indicated percentiles of the tumor size distribution. Each statistic is normalized to the tumor size at the corresponding percentile of the sg*Inert* distribution. Asterisks denote a statistically significant difference between young and aged (two-sided FDR-adjusted *P* < 0.05; *P* = 6.0 × 10^−4^ for 90th percentile, all other *P* < 1.0 × 10^−4^). Error bars indicate 95% confidence intervals around the point estimate of the test statistic (center of bars). *P* values and 95% confidence intervals were calculated using nested bootstrap resampling across 22 young and 20 aged mice. Sig., significant; NS, not significant. Source Data

**Extended Data Fig. 1:**
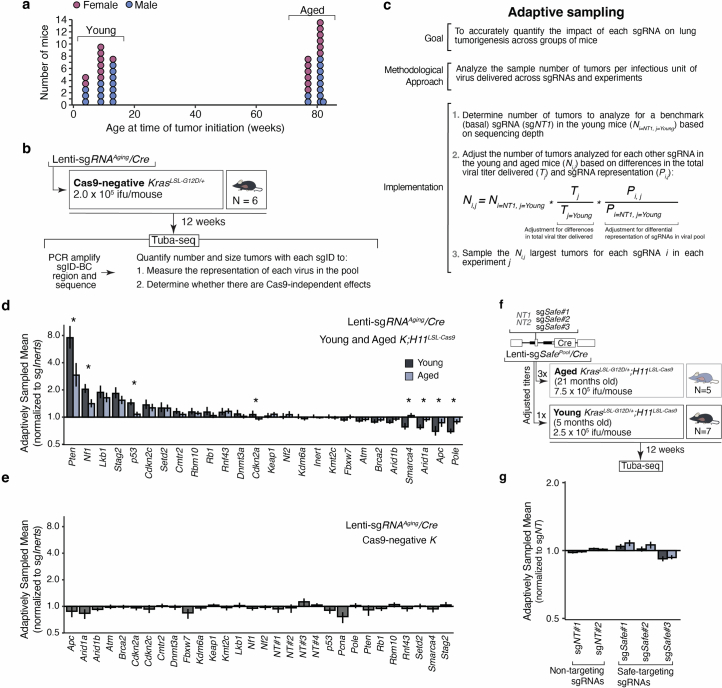
Aging impacts the effects of tumor suppressor gene inactivation in *K*;*H11*^*LSL-Cas9*^ mice. **a**. Ages of *Kras*^*LSL-G12D/+*^;*H11*^*LSL-Cas9*^ (*K*;*H11*^*LSL-Cas9*^) mice transduced with Lenti-sg*RNA*^*Aging*^/*Cre* (N = 22 young, 20 aged). Each dot is a mouse. Color denotes sex. **b**. Schematic of tumor initiation in *Kras*^*LSL-G12D/+*^ (Cas9-negative) mice with Lenti-sg*RNA*^*Aging*^/*Cre* to determine the representation of Lenti-sgRNA/*Cre* vectors. **c**. Explanation of the adaptive sampling method used to assess the impact of Lenti-sgRNA/Cre vectors (see Methods). **d**. Adaptively sampled mean size (ASM) of each tumor genotype normalized to the ASM of sg*Inert* tumors in the young and aged cohorts of *K*;*H11*^*LSL-Cas9*^ mice transduced with Lenti-sg*RNA*^*Aging*^/*Cre*. ASM is a summary metric that integrates the impact of inactivating each gene on tumor size and number. Line at x = 1 indicates no effect on tumorigenesis. Stars denote a statistically significant difference between young and aged (two-sided FDR-adjusted p value < 0.05. P = 0.0089, 0.029 for *Cdkn2a* and *Apc*, p < 10^−4^ for *Pten*, *Nf1*, *p53*, *Smarca4*, *Arid1a* and *Pole*). P values and confidence intervals were calculated using nested bootstrap resampling across 22 young and 20 aged mice. Genes are ordered by ASM in the young cohort. **e**. ASM relative to sg*Inert* vectors for sgRNAs in Lenti-sg*RNA*^*Aging*^/*Cre* in Cas9-negative *Kras*^*LSL-G12D/+*^ mice. Confidence intervals were calculated using nested bootstrap resampling across 6 mice. **f**. Initiation of KRAS-driven lung tumors in young and old *Kras*^*LSL-G12D/+*^;*R26*^*LSL-Tomato*^ mice with pooled non-targeting and safe-targeting Lenti-sgRNA/*Cre* vectors (Lenti-sg*Safe*^*Pool*^/*Cre*) to evaluate the impact of Cas9-induced dsDNA breaks on tumor initiation and growth. **g**. ASM of tumors initiated with each vector normalized to the ASM of sg*NT* tumors in the young and old cohorts of *Kras*^*LSL-G12D/+*^;*H11*^*LSL-Cas9*^ mice transduced with Lenti-sg*Safe*^*Pool*^/*Cre*. Line at x = 1 indicates no effect on tumorigenesis. No sgRNAs had significantly different effects between young and old (two-sided FDR-adjusted p value < 0.05; p = 0.41, 0.41, 0.25, 0.25, 0.42 for sg*NT#1*, sg*NT#2*, sg*Safe#1*, sg*Safe#2*, and sg*Safe#3*). P values and confidence intervals were calculated using nested bootstrap resampling across 7 young and 5 old mice. **d**,**e**,**g**: Errors bars indicate 95% confidence intervals around the point estimate of the test statistic (center of bars). Source Data

Alterations in tumor suppressor genes are ubiquitous in cancer and impact cellular processes that are critical to both cancer development and aging^25–28^. Integration of multiplexed CRISPR-based somatic genome engineering and tumor barcoding enables precise quantification of the growth of many genotypes of tumors in parallel^18,19,29^. To quantify the impact of aging on tumor suppressor gene function, we generated a pool of barcoded lentiviral vectors encoding *Cre* and targeting 25 known or putative tumor suppressor genes as well as ‘sg*Inert*’ non-targeting control vectors and a single-guide RNA (sgRNA) targeting an essential gene (*Pcna*) (Lenti-sg*RNA*^*Aging*^*/Cre*; Fig. 2a)^19^. The tumor suppressor genes included those with strong functional effects on initiation and/or growth in young mice^18,19^ and genes that are frequently mutated in human lung tumors^25,28,30^. We initiated lung tumors with Lenti-sg*RNA*^*Aging*^*/Cre* in young and aged *Kras*^*LSL-G12D/+*^(*K*)*;H11*^*LSL-Cas9*^ mice, as well as in a cohort of Cas9-negative *K* mice. Tuba-seq was performed on tumor-bearing lungs to quantify the size of each tumor of each genotype (Fig. 2b and Extended Data Fig. 1a,b). Analysis of all tumors as well as just those initiated with sg*Inert* vectors (which are driven solely by oncogenic KRAS) confirmed that tumor burden, tumor number, and size were all reduced in the aged mice (Supplementary Fig. 4a–e). Principal-component analysis of the number and size of tumors with each sgRNA relative to those with inert sgRNAs distinguished young and aged mice, suggesting that aging alters the spectrum of tumor-suppressive effects consistently across mice (Fig. 2c).

To quantify changes in the effects of inactivating each tumor suppressor gene with age, we sampled the same number of tumors per infectious unit of virus delivered for each sgRNA in each age group and calculated the log-normal mean size of these adaptively sampled tumors relative to sg*Inert* tumors (adaptively sampled mean (ASM); Methods and Extended Data Fig. 1c). The effects of tumor suppressor gene inactivation were qualitatively similar in the young and aged mice; with the exception of *Smarca4*, which was detrimental to tumor growth only in young mice, the genes that increased tumorigenesis when inactivated in young mice also increased tumorigenesis in aged mice (Fig. 2d). Notably, age reduced the average magnitude of effect of tumor suppressor gene inactivation (*P* = 2.3 × 10^−14^ for deviation of best fit line from equal effect line), with the impacts of several well-established lung tumor suppressor genes, including *Pten*, *p53* and *Nf1* significantly reduced in aged mice (Fig. 2d,e and Extended Data Fig. 1d,e). Conversely, the impacts of inactivating several other canonical tumor suppressor genes, including *Setd2*, *Stag2* and *Rbm10* were very similar in young and aged mice (Extended Data Fig. 1d,e).

Given the importance of p53 and Cdkn2a in the induction of senescence, it is notable that the tumor-suppressive function of these genes was not enhanced with age, as would be expected if senescence constrained tumor development in aged mice. While these findings do not rule out a role for senescence in repressing tumorigenesis in aged mice, these genetic experiments suggest that senescence may not be an important contributor to the reduction in tumorigenesis with age.

Cutting with Cas9 induces double-stranded DNA breaks, which can be cytotoxic^31^. To assess whether there is differential sensitivity to dsDNA breaks with age that could confound these analyses, we compared tumors initiated with safe-targeting and non-targeting Lenti-*Cre* vectors (Extended Data Fig. 1f). There was no difference in the effect of safe-targeting sgRNAs between young and aged mice, indicating that aged lung epithelial cells are not more sensitive to Cas9-induced dsDNA breaks (Extended Data Fig. 1g). Taken together, these data suggest that while tumor suppressor function is broadly conserved with age, the molecular changes associated with aging lessen the importance of specific tumor- suppressor pathways in constraining tumorigenesis.

**Extended Data Fig. 2:**
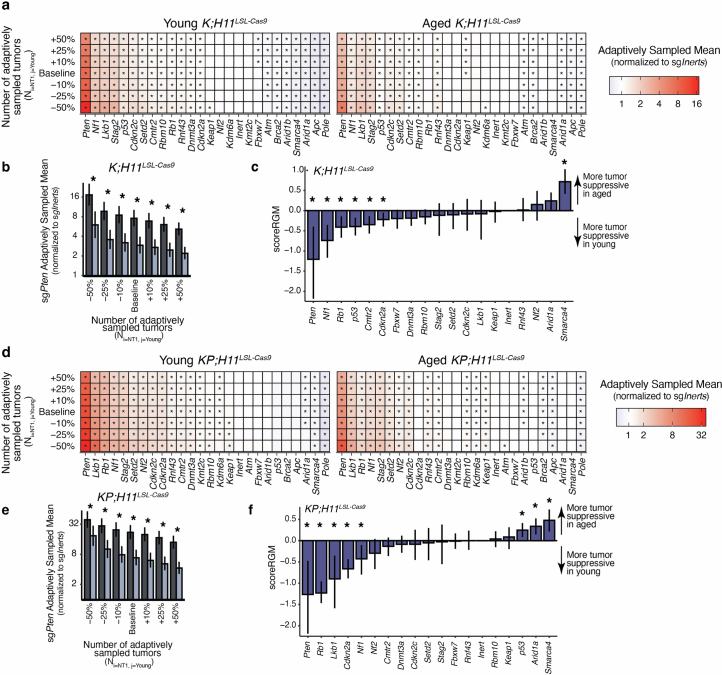
Differential effect of *Pten* inactivation with age is robust to variation in number of tumors adaptively sampled and statistics used to quantify impact of age on tumor suppressor function. **a**. Adaptively sampled mean (ASM) tumor sizes normalized to sg*Inert* for all tumor genotypes in young and old *K*;*H11*^*LSL-Cas9*^ mice transduced with Lenti-sg*RNA*^*Aging*^/*Cre*, varying the number of tumors adaptively sampled by the indicated percentages. Stars denote a significant impact on ASM (two-sided FDR-corrected p < 0.05). This sensitivity analysis demonstrates stability in ASMs across a range of adaptively sampled tumor numbers. **b**. The impact of sg*Pten* on tumorigenesis is greater in young than old *K*;*H11*^*LSL-Cas9*^ mice across a range of adaptively sampled tumor numbers. Stars indicate a differential effect with age (FDR-adjusted two-sided p value < 0.05; p = 4.0×10^−4^ for −50%, all other p < 1.0×10^−4^). For **a**-**b**: ‘Baseline’ corresponds to the analyses shown in Fig. 2. **c**. Differential effects of tumor suppressor inactivation on tumor size with age quantified by scoreRGM (Li et al.) in *K*;*H11*^*LSL-Cas9*^ mice transduced with Lenti-sg*RNA*^*Aging*^/*Cre*. Stars indicate differential effect with age (FDR-adjusted two-sided p value < 0.05; p = 0.028, 0.011, 0.028, 0.011, 0.011, 0.036 for *Pten*, *Nf1*, *Rb1*, *p53*, *Cmtr2*, and *Cdkn2a*, p < 10^−4^ for *Smarca4*.). **d**. ASM tumor sizes normalized to sg*Inert* for all tumor genotypes in young and old *K*;*P53* ^*flox/flox*^;*H11*^*LSL-Cas9*^ (*KP*;*H11*^*LSL-Cas9*^) mice transduced with Lenti-sg*RNA*^*Aging*^/*Cre*, varying the number of tumors adaptively sampled by the indicated percentages. Stars denote a significant impact on ASM (two-sided FDR-corrected p < 0.05). This sensitivity analysis demonstrates stability in ASMs across a range of adaptively sampled tumor numbers. **e**. The impact of sg*Pten* on tumorigenesis is greater in young than old *KP*;*H11*^*LSL-Cas9*^ mice across a range of adaptively sampled tumor numbers. Stars indicate a differential effect with age (FDR-adjusted two-sided p value < 0.05; p = 1.7×10^−2^ for −50%, all other p < 1.0×10^−4^). For **d**-**e**: ‘Baseline’ corresponds to the analyses shown in Fig. 3. **f**. Differential effects of tumor suppressor inactivation on tumor size with age quantified by scoreRGM (Li et al.) in *KP*;*H11*^*LSL-Cas9*^ mice transduced with Lenti-sg*RNA*^*Aging*^/*Cre*. Stars indicate differential effect with age (FDR-adjusted two-sided p value < 0.05; p = 0.019, 0.012, 0.043, 0.028, 0.0094, 0.0018 for *Pten*, *Lkb1*, *Nf1*, *p53*, *Arid1a* and *Smarca4*, p < 10^−4^ for *Rb1* and *Cdkn2a*.) **a**-**c**: N = 22 young and 20 old *K*;*H11*^*LSL-Cas9*^ mice. **d**-**f**: N = 16 young and 25 old *KP*;*H11*^*LSL-Cas9*^ mice. **All panels**: Error bars indicate 95% confidence intervals around the point estimate of the test statistic (center of bars). P values and confidence intervals were calculated using nested bootstrap resampling. Source Data

Although PTEN suppressed lung tumorigenesis in both young and aged mice, *Pten* inactivation increased tumor burden >2.5 times more in young than in aged mice (bootstrapped *P* < 1 × 10^−4^; Fig. 2e and Extended Data Fig. 1d). This reduction stands out even in the context of the broad dampening of effects with age; in young mice inactivation of *Pten* increased tumorigenesis nearly four times more than inactivation of the next strongest tumor suppressor, whereas in aged mice it was less than twice as strong as the next strongest tumor suppressor (Extended Data Fig. 1d). Comparing the size of sg*Pten* and sg*Inert* tumors at various percentiles of the tumor size distribution in young and aged mice also consistently showed that age reduces the impact of *Pten* inactivation (Fig. 2f). To ensure the robustness of these findings, we repeated our analyses varying the number of tumors sampled per infectious unit of virus and analyzing the impacts of tumor suppressor inactivation separately in males and females. In addition, we used an alternative statistical method to compare the effects of tumor suppressors between young and aged mice that scales the number of tumors analyzed based on both a genotype-independent effect of age (estimated by comparing the sg*Inert* tumor size distributions for young and aged mice) and the representation of each Lenti-sgRNA/Cre vector (‘ScoreRGM’, score relative geometric mean; Methods). These analyses recapitulated that the effects of most tumor suppressor genes are weakened with age, and consistently found that *Pten* inactivation had the most dramatically age-dependent effect irrespective of sex (Extended Data Fig. 2a–c and Supplementary Fig. 5a–c).

**Extended Data Fig. 3:**
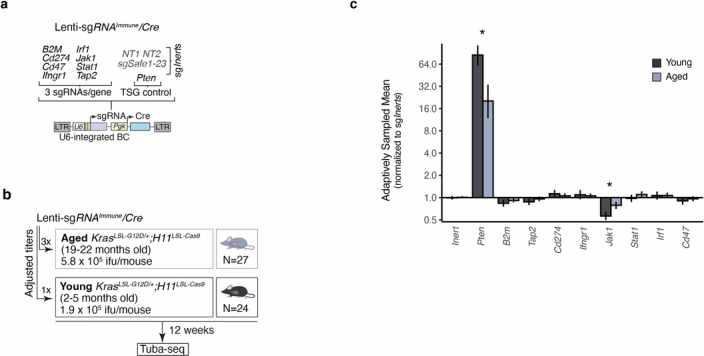
The impacts of inactivating genes involved in the anti-tumor immune response on tumor growth are weak and not strongly impacted by age. **a**. Barcoded Lenti-sgRNA/*Cre* vectors in the Lenti-sg*RNA*^*Immune*^/*Cre* pool include vectors targeting key nodes of the anti-tumor immune response, the tumor suppressor gene *Pten*, and both non-targeting and safe-targeting inert vectors. **b**. Initiation of KRAS-driven lung tumors in young and old *Kras*^*LSL-G12D/+*^;*R26*^*LSL-Tomato*^ mice with Lenti-sg*RNA*^*Immune*^/*Cre* to evaluate the impact of modulating anti-tumor immune responses on tumor initiation and growth in young and old mice. Age and number of mice are indicated. **c**. Adaptively sampled mean size (ASM) of tumors initiated with each vector normalized to the ASM of sg*Inert* tumors in the young and old cohorts of *Kras*^*LSL-G12D/+*^;*H11*^*LSL-Cas9*^ mice transduced with Lenti-sg*RNA*^*Immune*^/*Cre*. ASM is a summary metric of tumor fitness that integrates the impact of inactivating each gene on tumor size and number. Line at x = 1 indicates no effect on tumorigenesis. Stars denote a statistically significant difference (two-sided FDR-adjusted p value < 0.05) between young and old. Error bars indicate 95% confidence intervals around the point estimate of the test statistic (center of bars). P values and confidence intervals were calculated using nested bootstrap resampling across 24 young and 27 old mice. p = 1.0×10^−3^ for *Jak1* and p < 1.0×10^−4^ for *Pten*. Source Data

Changes in the immune system with age are a potentially important mechanistic linkage between aging and tumorigenesis that could contribute to both the overall reduction in tumorigenesis with age, and to the differential impacts of tumor suppressor gene inactivation. To assess the impact of immune aging on tumorigenesis in this model, we initiated tumors in young and aged *K;H11*^*LSL-Cas9*^ mice using a barcoded Lenti-sgRNA/Cre pool containing vectors targeting key nodes of the antitumor immune response, including antigen presentation (*B2m* and *Tap2*), IFNGR1 signaling (*Ifngr1*, *Jak1*, *Stat1* and *Irf1*) and immune tolerance (*Cd274* and *Cd47*) and performed Tuba-seq after tumor development (Extended Data Fig. 3a,b). Inactivating most of these genes had no effect or a very weak effect in either young or aged mice, suggesting that tumors in this model are not strongly immunogenic and that immune aging (at least through the pathways that we targeted) is not an important driver of the observed phenotypes (Extended Data Fig. 3c).

### Age impacts tumor suppression in P53-deficient lung tumorigenesis

**Fig. 3:**
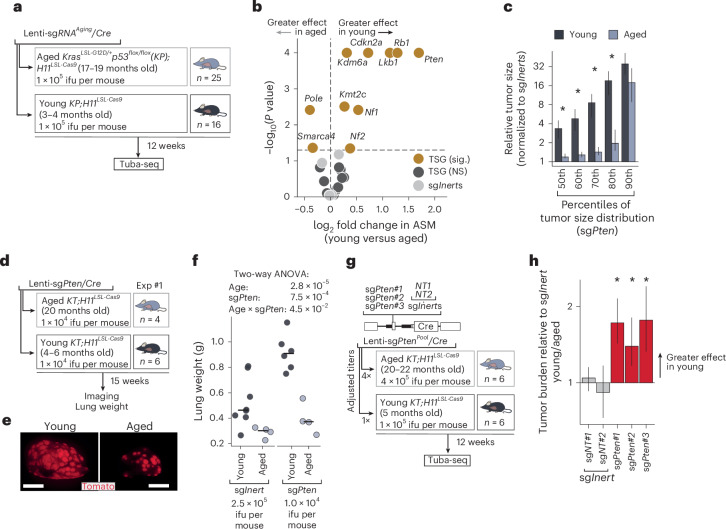
Validation of age-dependent differences in tumor suppression by *Pten.* **a**, Initiation of lung tumors in young and aged *KP;H11*^*LSL-Cas9*^ mice with Lenti-sg*RNA*^*Aging*^*/Cre*. Genotype, ages, lentiviral titer and mouse number are indicated. Mice were analyzed after 12 weeks of tumor growth. See Extended Data Fig. 4a for exact ages and sexes. **b**, Volcano plot showing fold change in the ASM of tumors in the young versus aged *KP;H11*^*LSL-Cas9*^ cohorts. ASM is a summary metric of tumor fitness that integrates the impact of inactivating each gene on tumor size and number. Reported ASMs for each genotype are normalized to the ASM of sg*Inert* tumors. TSGs with a significantly different impact in young and aged mice (two-sided FDR-adjusted *P* value < 0.05) are highlighted in gold and labeled. sg*Inert* vectors are highlighted in light gray. *P* values were calculated using nested bootstrap resampling across 16 young and 25 aged mice. **c**, Adaptively sampled percentile sizes of tumors with sg*Pten* vectors in young and aged *KP;H11*^*LSL-Cas9*^ mice transduced with Lenti-sg*RNA*^*Aging*^*/Cre* pool. Each statistic is normalized to the tumor size at the corresponding percentile of the sg*Inert* distribution. Asterisks denote a statistically significant difference between young and aged (two-sided FDR-corrected *P* < 0.05; *P* = 3.5 × 10^−2^ for 90th percentile, all other *P* < 1.0 × 10^−4^). Error bars indicate 95% confidence interval around the point estimate of the test statistic (center of bars). *P* values and 95% confidence intervals were calculated using nested bootstrap resampling across 16 young and 25 aged mice. **d**, Schematic of tumor initiation in young and aged mice with Lenti-sg*Pten/Cre*. See Extended Data Fig. 4e for exact ages and sexes. **e**, Representative Tomato fluorescence images of lungs of young and aged mice with tumors initiated with Lenti-sg*Pten/Cre*. Scale bars, 5 mm. **f**, Lung weights of young and aged mice transduced with Lenti-sg*Inert/Cre* (*n* = 8 young and 4 aged) and Lenti-sg*Pten/Cre* (*n* = 6 young and 4 aged). Two-way analysis of variance indicates that there is a statistically significant interaction between the effects of age and *Pten* inactivation on lung weight (*F*(1,18) = 4.66, *P* = 0.45). See Extended Data Fig. 4f for post hoc analysis. **g**, Schematic of tumor initiation in young and aged mice with Lenti-sg*Pten*^*Pool*^*/Cre*. See Extended Data Fig. 4e for exact ages and sexes. **h**, Tumor burden per sgRNA relative to sg*Inert* tumor burden, in young relative to aged mice transduced with Lenti-sg*Pten*^*Pool*^*/Cre*. Stars denote a significant differential effect with age (two-sided FDR-corrected *P* < 0.05; *P* < 1.0 × 10^−4^ for sg*Pten#1* and sg*Pten#2*, *P* = 1.3 × 10^−3^ for sg*Pten#2*). Error bars indicate 95% confidence interval around the point estimate of the test statistic (center of bars). *P* values and 95% confidence intervals were calculated using nested bootstrap resampling across six young and six aged mice. Source Data

**Extended Data Fig. 4:**
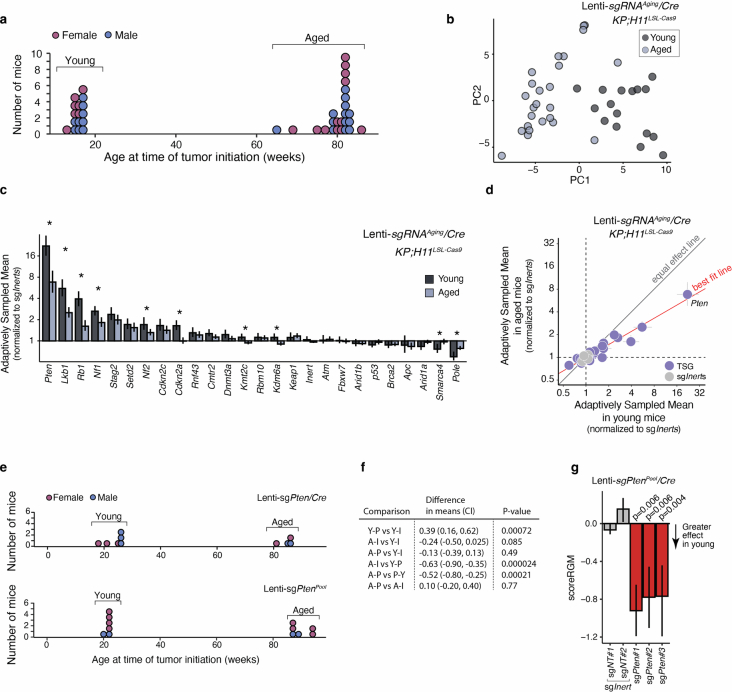
Validation of changes in tumor suppressor function with age. **a**. Ages of *KP*;*H11*^*LSL-Cas9*^ mice transduced with Lenti-sg*RNA*^*Aging*^/*Cre*. Each dot is a mouse; color denotes sex. **b**. Principal-component analysis of tumor-suppressive effects performed within each mouse reveals separation of young and old *KP*;*H11*^*LSL-Cas9*^ mice transduced with Lenti-sg*RNA*^*Aging*^/*Cre*. Each dot is a mouse. **c**. Adaptively sampled mean size (ASM) of each tumor genotype normalized to the ASM of sg*Inert* tumors in the young and old *KP*;*H11*^*LSL-Cas9*^ mice cohorts transduced with Lenti-sg*RNA*^*Aging*^/*Cre*. ASM is a summary metric that integrates the impact of inactivating each gene on tumor size and number. Line at x = 1 indicates no effect on tumorigenesis. Stars indicate differential effect withe age (FDR-adjusted two-sided p < 0.05; p = 0.0030, 0.037, 0.0023, 0.035, 0.0030 for *Nf1*, *Nf2*, *Kmt2c*, *Smarca4*, and *Pole*, p < 10^−4^ for *Pten*, *Lkb1*, *Rb1*, *Cdkn2a*, and *Kdm6a*). Error bars indicate 95% confidence intervals around the point estimate of the test statistic (center of bars). P values and confidence intervals were calculated using nested bootstrap resampling across 16 young and 25 old mice. **d**. ASM of each tumor genotype normalized to the ASM of sg*Inert* tumors in the young (x-axis) versus old (y-axis) *KP*;H11^LSL-Cas9^ mice cohorts transduced with Lenti-sg*RNA*^*Aging*^/*Cre*. **e**. Ages of young and old mice transduced with Lenti-sg*Pten*/*Cre* in Exp #1 (top) and with Lenti-sg*Pten*^*Pool*^/*Cre* pool (bottom). Each dot is a mouse; color denotes sex. **f**. Post hoc analysis with Tukey’s test corresponding to two-way ANOVA in Fig. 3f. Y-P: young sg*Pten*; Y-I: young sg*Inert*; O-P: old sg*Pten*; O-I: old sg*Inert*; CI: lower and upper values of 95^th^ percentile confidence interval. **g**. Differential effects of each sgRNA in Lenti-sg*Pten*^*Pool*^/*Cre* with age as quantified by scoreRGM (Li et al.). Stars indicate differential effect with age. Error bars indicate 95% confidence intervals around the point estimate of the test statistic (center of bars). FDR-adjusted two-sided p values and confidence intervals were calculated using nested bootstrap resampling across 6 young and 6 old mice. Source Data

*TP53* is the most frequently mutated tumor suppressor gene in lung adenocarcinoma and is implicated in aging^25,28,30,32,33^. Previous data from young mice suggest that some tumor suppressor genes function differently in the context of p53 deficiency^34^; therefore, we initiated tumors with Lenti-sg*RNA*^*Aging*^*/Cre* in young and aged *K;p53*^*flox/flox*^*;H11*^*LSL-Cas9*^ mice (*KP;H11*^*LSL-Cas9*^) in which all tumors will be p53-deficient (Fig. 3a and Extended Data Fig. 4a). After 12 weeks of tumor growth, we performed Tuba-seq on bulk-tumor-bearing lungs and compared the ASM tumor sizes for each genotype in young and aged mice. This experiment recapitulated several key patterns identified in the *K;H11*^*LSL-Cas9*^ mice, including both the qualitative similarity in tumor-suppressive effects between young and aged mice and the broad dampening of the impacts of tumor suppressor gene inactivation in aged mice (Fig. 3b and Extended Data Fig. 4b–d). All tumor suppressors with reduced effects in aged *K;H11*^*LSL-Cas9*^ mice also had reduced effects in aged *KP;H11*^*LSL-Cas9*^ mice (with the exception of *p53*, which as expected had no impact in either young or aged *KP;H11*^*LSL-Cas9*^ mice). *Pten* inactivation again had a notable age-dependent effect on tumorigenesis (Fig. 3b,c and Extended Data Fig. 4c,d).

There was also consistency in which tumor suppressor genes did not interact with age within p53-proficient and p53-deficient tumors. Age did not impact the effects of inactivation of *Stag2, Setd2, Cdkn2c* and *Rbm10* in either context, further suggesting that age interacts with specific tumor suppressor pathways as opposed to generically weakening the effects of driver mutations (Fig. 3b and Extended Data Fig. 4c). Sensitivity analyses again showed that these effects are present in both males and females and are robust to variation in the number of tumors sampled and the use of an alternative statistical method (Extended Data Fig. 2d–f and Supplementary Fig. 5d–f).

### Age weakens the tumor-suppressive effect of PTEN

*Pten* inactivation had the greatest reduction in effect with age among the tumor suppressor genes we assayed in both oncogenic KRAS and KRAS;P53-deficient lung tumors. To validate the impact of aging on the tumor-suppressive function of PTEN outside of a pooled setting, we initiated tumors in young and aged *Kras*^*LSL-G12D/+*^*;Rosa26*^*LSL-tdTomato*^*;H11*^*LSL-Cas9*^ (*KT;H11*^*LSL-Cas9*^) mice with a lentiviral vector encoding Cre recombinase and a *Pten*-targeting sgRNA (‘sg*Pten*’ vector; Fig. 3d and Extended Data Fig. 4e). Aged mice had visibly fewer tumors and dramatically lower lung weights (Fig. 3e,f). Notably, the reduction in lung weight with age in mice transduced with the sg*Pten* vector exceeded that of mice transduced with the sg*Inert* vectors (two-way analysis of variance (ANOVA) *P* = 0.045 for sg*Pten*-by-age interaction term; Fig. 3f and Extended Data Fig. 4f). Pten-deficient tumors in young and aged mice were of similar grades and had not progressed to a malignant HMGA2^positive^ state (Supplementary Fig. 6a–c).

To further validate that age reduces the tumor-suppressive function of PTEN and confirm that this result is driven by on-target effects, we generated a pool of barcoded Lenti-sgRNA/Cre vectors containing three distinct sgRNAs targeting *Pten* as well as two sg*Inert* control vectors (Lenti-sg*Pten*^*Pool*^*/Cre*). We initiated tumors with Lenti-sg*Pten*^*Pool*^*/Cre* in young and aged *KT;H11*^*LSL-Cas9*^ mice and performed Tuba-seq on bulk tumor-bearing lungs (Fig. 3g and Extended Data Fig. 4e). We calculated the impact of each sgRNA on tumor burden relative to the sg*Inert* vectors and compared this metric between young and aged mice. In addition, we calculated scoreRGM^35^ for each sgRNA. All three *Pten*-targeting sgRNAs had a greater impact on tumor burden in young mice than in aged mice by both metrics (Fig. 3h and Extended Data Fig. 4g). Thus, the tumor-suppressive effect of *Pten* is weakened with age across three distinct sgRNAs and across four separate experiments in young and aged mice.

### Age has a continued impact on the gene expression state of neoplastic cells

**Fig. 4:**
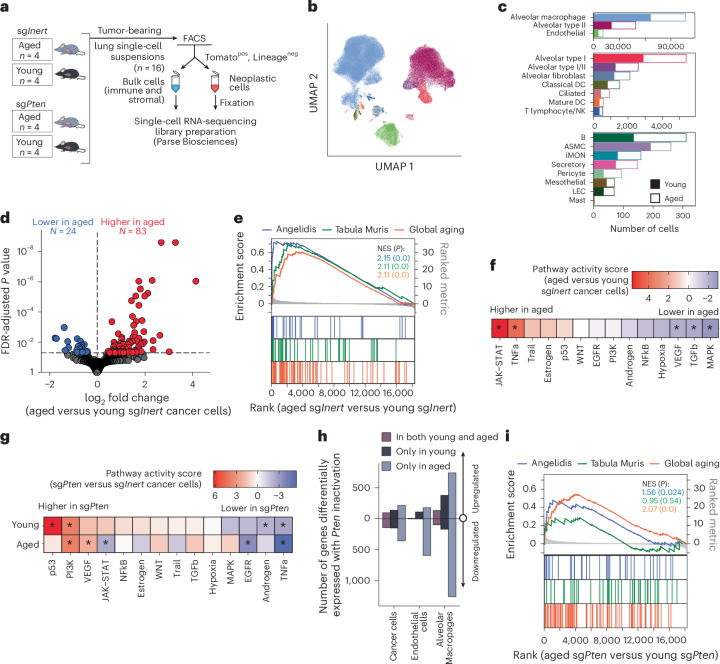
Aging alters cancer cell state and the impacts of *Pten* inactivation. **a**, scRNA-seq of cells from tumor-bearing lungs of young and aged *KT;H11*^*LSL-Cas9*^ mice transduced with Lenti-sg*Inert/Cre* or Lenti-sg*Pten/Cre* vectors. To enrich for cancer cells, whole-lung single-cell suspensions were divided into a cancer cell fraction and a bulk fraction containing all other live cells by fluorescence-activated cell sorting (FACS). Lineage, markers of non-epithelial lineages (CD45, CD31, Ter119 and F4/80). **b**, Uniform manifold approximation and projection (UMAP) embedding of cells colored by cell type. Mapping of colors to cell types is indicated in **c**. **c**, Counts per cell type by age. DC, dendritic cell; ASMC, airway smooth muscle cell; iMON, inflammatory monocytes; NK, natural killer; LEC, lymphatic endothelial cell. Cell type definitions are from Guo et al.^73^ (Methods). **d**, Volcano plot showing changes in gene expression with age in cancer cells from the sg*Inert* samples. Differentially expressed genes (Benjamini–Hochberg-corrected two-sided Wald test *P* < 0.05) are highlighted in color. **e**, Gene expression signatures of normal aging are enriched with age in KRAS-driven lung cancer cells. Angelidis, genes with positive log fold change with aging and adjusted *P* value < 0.05 in Angelidis et al.^38^; Tabula Muris, lung type II pneumocyte aging gene set from the Tabula Muris Consortium; Global Aging, global aging genes upregulated with age from Zhang et al.^42^. NES, normalized enrichment score. *P* values calculated by permutation test where observed enrichment scores are compared to scores generated by random permutation of gene ranks and are FDR corrected. **f**, Pathway activity scores comparing aged to young cancer cells from mice with sg*Inert* tumors. Positive scores (red) indicate that a pathway is more active with age; negative scores (blue) indicate that a pathway is less active with age. Asterisks denote that a pathway is differentially activated with age (*P* < 0.05, PROGENy multivariate linear model; Methods). **g**, Pathway activity scores comparing PTEN-deficient (sg*Pten*) to wild-type (sg*Inert*) for young and aged cancer cells. Positive scores (red) indicate that a pathway is more active with *Pten* inactivation; negative scores (blue) indicate that a pathway is less active with *Pten* inactivation. Asterisks denote that a pathway is differentially activated with *Pten* inactivation (*P* < 0.05, PROGENy multivariate linear model; Methods). **h**, Numbers of genes differentially expressed with *Pten* inactivation in indicated cell types, stratified by whether genes were up/downregulated, and by whether effect is specific to young or aged cells, or is common to both ages. **i**, Enrichment of gene expression signatures of normal aging (as in **e**) are weakened in *Pten*-deficient lung tumors. Source Data

**Extended Data Fig. 5:**
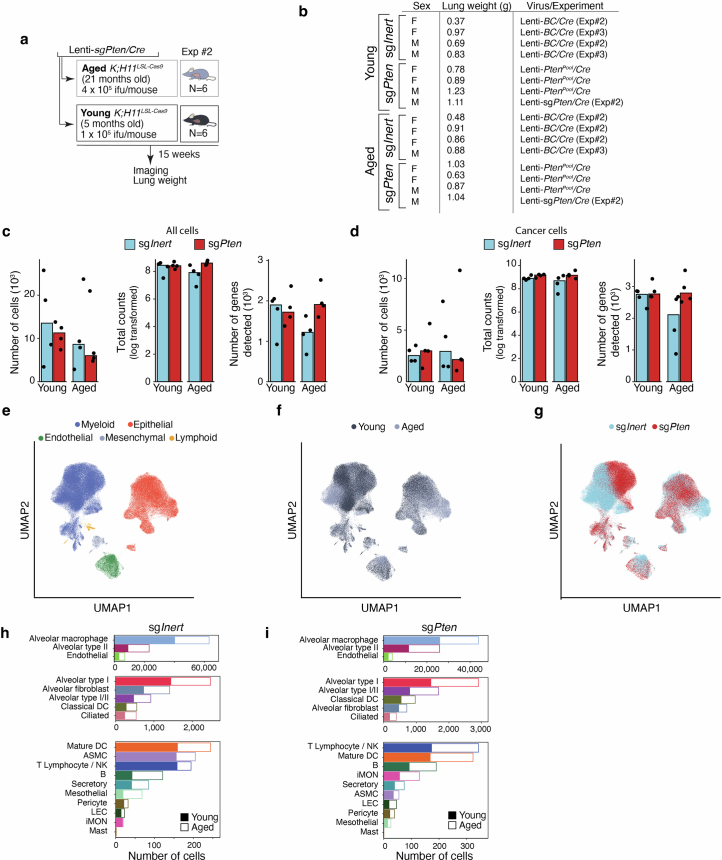
Single-cell RNA sequencing characterizes impacts of aging and *Pten* inactivation on tumor-bearing lungs. **a**. Schematic of tumor initiation for additional experiment to generate lung samples with *Pten*-deficient tumors. **b**. Table describing characteristics of samples used to generate scRNA-sequencing data. **c**. Total number of cells, median counts per cell, and median number of genes per cell detected per sample after quality control. Each dot is a sample. Tops of bars indicate median values across N = 4 young and 4 old mice with sg*Inert* tumors and N = 4 young and 4 old mice with sg*Pten* tumors. **d**. Number of cancer cells, median counts per cancer cell, and median number of genes per cancer cell after quality control. Each dot is a sample. Tops of bars indicate median values across N = 4 young and 4 old mice with sg*Inert* tumors and N = 4 young and 4 old mice with sg*Pten* tumors. **e**-**g**. Uniform Manifold Approximation and Projection (UMAP) embedding of cells colored by lineage identity (**e**), age (**f**), and tumor genotype (**g**). **h**,**i**. Counts per cell type by age for sg*Inert* (**h**) and sg*Pten* (**i**) samples. DC: dendritic cells, ASMC: airway smooth muscle cells, iMON: inflammatory monocytes, NK: natural killer cells, LEC: lymphatic endothelial cells. Source Data

**Extended Data Fig. 6:**
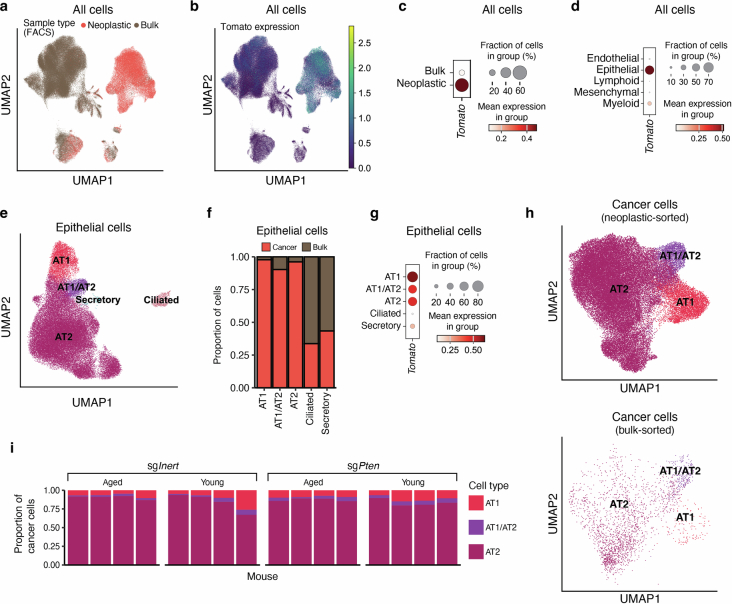
Definition of neoplastic cell populations from single-cell RNA sequencing data. **a**. Uniform Manifold Approximation and Projection (UMAP) embedding of cells colored by whether they were sorted as ‘neoplastic cells’ or ‘bulk cells’ (see Fig. 4a and Methods). **b**-**c**. *Tomato* is frequently and highly expressed in cells sorted as neoplastic cells, confirming that sample sorting and preparation worked as expected. **b**, UMAP embedding of cells colored by *Tomato* expression. **c**, Quantification of *Tomato* expression by sample type. **d**. *Tomato* is primarily expressed in cells annotated as epithelial-like, consistent with the expected phenotype of lung adenoma and adenocarcinoma cells. **e**. UMAP embedding of epithelial-like cells colored by annotated cell type. **f**,**g**. The majority of cells annotated as AT1-, AT2-, and AT1/AT2-like cells were sorted as neoplastic cell samples by FACS (**f**) and express *Tomato* (**g**), suggesting that these cell types consist primarily of cancer cells in our dataset. **h**. UMAP embedding of cancer cells (that is AT1-, AT2- and AT1/AT2-like) stratified by whether cells were sorted into neoplastic cell samples (top) or bulk cell samples (bottom). AT1/AT2/AT1.AT2-like cells sorted into bulk cell samples have a higher potential to be untransduced normal lung epithelial cells and were thus excluded from downstream analyses including differential gene expression analysis and age signature scoring (see Methods). **i**. Breakdown of cancer cells by alveolar epithelial cell type for each sample. Source Data

To assess changes in neoplastic cell state and the tumor microenvironment with age and gain insights into the age-dependent effect of PTEN, we performed single-cell RNA sequencing (scRNA-seq) on cells from the tumor-bearing lungs of young and aged *KT;H11*^*LSL-Cas9*^ mice with tumors initiated with Lenti-sg*Inert/Cre* (sg*Inert*) and Lenti-sg*Pten/Cre* (sg*Pten*) (*n* = 4 mice per age-genotype group; Fig. 4a and Extended Data Fig. 5a,b). We separately enriched for neoplastic cells and for all other stromal and immune cells by fluorescence-activated cell sorting (Fig. 4a and Methods). After quality controls, including in silico removal of transduced non-neoplastic cells, ~180,000 cells (~100,000 from young and ~80,000 from aged mice) were assigned to 19 expected cell types in the mouse lung^36,37^ (Fig. 4b,c, Extended Data Fig. 5c,d and Methods). Each cell type included cells from each age group and genotype, indicating that neither age nor *Pten* inactivation confounded cell type annotation (Fig. 4c and Extended Data Fig. 5e–i). Across the five major epithelial-like cell subtypes, *Tomato*-expressing cells were greatly enriched in alveolar type 2 (AT2), AT1/2- and AT1-like populations. We therefore focused on these cells as the neoplastic/cancer cell fraction (Extended Data Fig. 6a–i and Methods).

**Extended Data Fig. 7:**
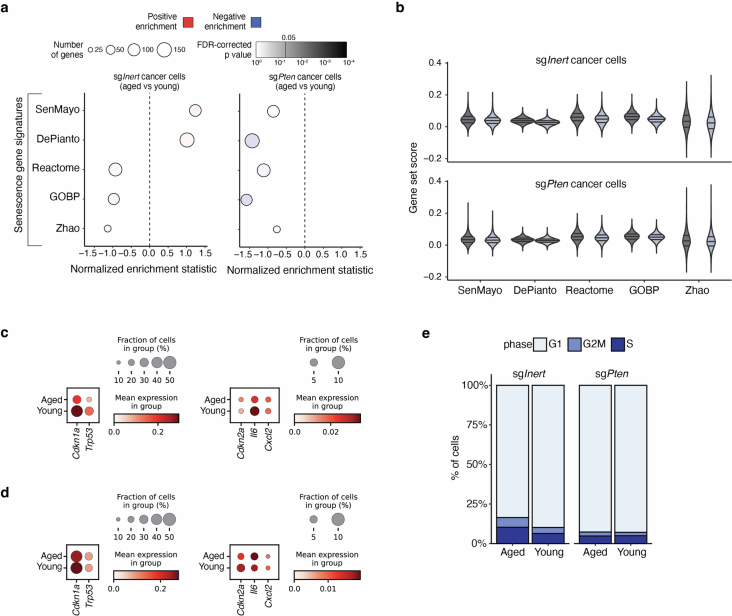
Transcriptomic signatures of senescence are not enriched in aged cancer cells. **a**. Transcriptomic signatures of senescence are not enriched with age in PTEN-proficient (sg*Inert*, left) or PTEN-deficient (sg*Pten*, right) oncogenic KRAS-driven lung cancer cells. Positive enrichment statistic (red) indicates that a signature is enriched in old cancer cells; negative statistic (blue) indicates that a signature is enriched in young cancer cells. Shading indicates significance from permutation test where observed enrichment scores are compared to scores generated by random permutation of gene ranks; no signatures were significantly enriched (FDR-corrected p < 0.05) in either context. SenMayo: cross-tissue and species senescence signature from Saul et al., DePianto: lung epithelial senescence signature from Depianto et al., Reactome: Reactome cellular senescence gene set; GOBP: Gene Ontology Biological Process cellular senescence gene set; Zhao: cross-cancer type senescence signature from Zhao et al. **b**. Per-cell scoring of gene expression signatures of senescence in cancer cells (see Methods), stratified by age and genotype. **c**,**d**. Canonical senescence and SASP genes are not consistently expressed more frequently or more highly in sg*Inert* (**c**) or sg*Pten* (**d**) cancer cells. Genes are split to facilitate visualization of less frequently expressed genes. **e**. Cell cycle phase assignments by age for PTEN-proficient (sg*Inert*, left) and PTEN-deficient (sg*Pten*, right) cancer cells. Source Data

Single-cell mapping efforts have revealed extensive transcriptomic changes with age^23,38–41^. Oncogenic transformation is a major departure from cellular homeostasis^15^. Thus, it is unclear whether cells in the tumors of aged mice would retain these canonical features of aging or would converge to a shared molecular state with young cancer cells. sg*Inert* neoplastic cells from tumors in young and aged mice were represented similarly within the major epithelial-like cell clusters, suggesting that age does not lead to dramatic shifts in cell identity (Extended Data Fig. 6i). However, pseudobulk comparison between young and aged neoplastic cells uncovered significantly differently expressed genes (Fig. 4d). Neoplastic cells from tumors that developed in aged mice (‘aged cancer cells’) had a striking enrichment for several signatures of lung epithelial^23,38^ and global aging^42^ when compared to neoplastic cells from tumors that developed in young mice (‘young cancer cells’), suggesting that oncogenic KRAS-driven lung tumorigenesis does not revert these fundamental changes (Fig. 4e). Consistent with our genetic results inactivating *p53* and *Cdkn2a*, signatures of senescence were not enriched in aged cancer cells, further suggesting that senescence is not responsible for the reduced growth of aged tumors^43–46^ (Extended Data Fig. 7a–e).

To further understand the impacts of age on the molecular state of cancer cells, we used PROGENy^47^ to infer changes in the activation of canonical signaling pathways with age. Of note, several pathways that could logically impact tumorigenesis were differentially active with age, including reduced MAPK signaling in aged cancer cells, which has a critical role in driving proliferation downstream of oncogenic KRAS (Fig. 4f). Thus, the impact of age on KRAS-driven lung tumors continues beyond initiation and the earliest stage of tumorigenesis.

### Age changes the transcriptomic effects of *Pten* inactivation on cancer cells and the tumor microenvironment

*Pten* inactivation has a highly age-dependent impact on tumor fitness (Figs. 2 and 3). To gain insight into the molecular basis of this genotype-by-age interaction, we compared the effects of *Pten* inactivation on the transcriptomes of young and aged cancer cells. Consistent with the function of PTEN as a canonical negative regulator of PI3K signaling^48^, this pathway was upregulated to a similar extent in both young and aged sg*Pten* cancer cells. However, changes in the activation of several other pathways were specific to a single age context (Fig. 4g). These results argue against a simple model in which age reduces the fitness benefit associated with *Pten* inactivation through curbing activation of the pathway that it suppresses, but instead suggest that aging alters the molecular consequences further downstream of PI3K activation or through PI3K-independent mechanisms.

**Extended Data Fig. 8:**
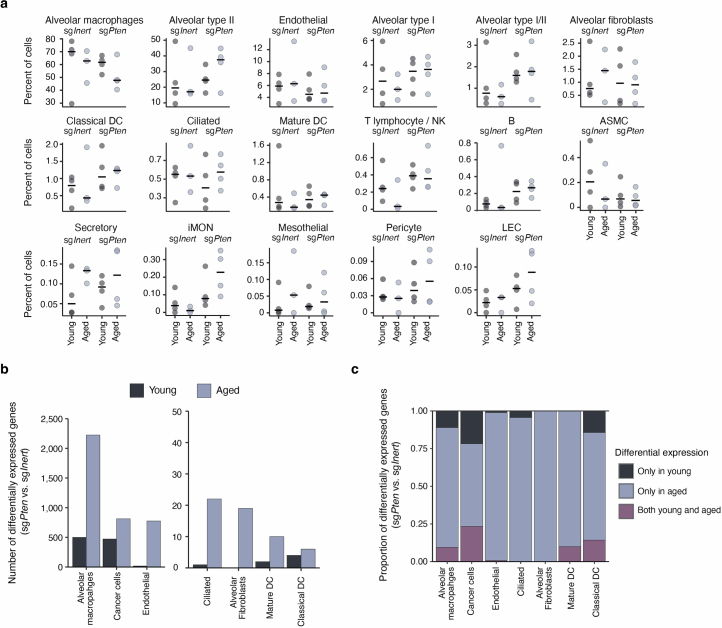
Age shapes the impact of *Pten* inactivation on the tumor microenvironment. **a**. Representation of cell types in single-cell RNA sequencing data by age and tumor genotype. Each dot is a mouse; bars indicate median values across N = 4 young and 3 old mice with sg*Inert* tumors and N = 4 young and 4 old mice with sg*Pten* tumors. Cell types are ordered by overall abundance in the dataset. The impacts of age and tumor genotype on the representation of each cell type were evaluated by two-way ANOVA. No effects were significant after FDR correction. **b**. Number of genes differentially expressed in mice with PTEN-deficient tumors (Benjamini–Hochberg-corrected two-sided Wald test p < 0.05) in indicated cell types from young and old mice in cell-type-resolved pseudobulk differential gene expression analysis. Only cell types with at least two pseudobulked samples (corresponding to at least 10 cells and at least 1,000 counts) per age-genotype category were included in the analysis. **c**. Proportion of genes for indicated cell types that that are differentially expressed with the growth of PTEN-deficient tumors specifically in young mice, specifically in old mice, or in both young and old mice. Genes differentially expressed in both young and old mice were expressed in concordant directions (that is overexpressed or underexpressed in both young and old mice with the exception of two genes in alveolar macrophages. DC: dendritic cells. Source Data

**Extended Data Fig. 9:**
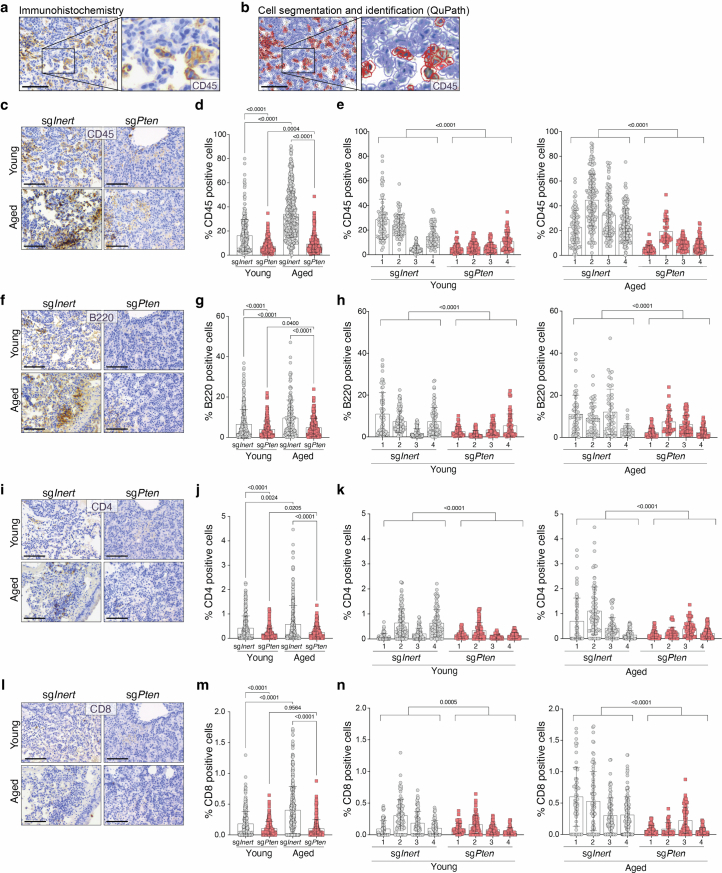
Quantification of tumor-infiltrating immune cells. **a**, **b**. Immunohistochemical staining for CD45 (**a**) and cell segmentation and idenfitication using QuPath (**b**). Example is the Young sg*Inert* section shown in panel **c**. Scale bars = 100 μM. **c**,**f**,**i**,**l**. Representative immunohistochemical stainings for CD45 (**c**), B220 (**f**), CD4 (**i**), and CD8 (**l**). Scale bars = 100 μM **d**,**g**,**j**,**m**. Each symbol represents a tumor. Bars are the mean of all tumors from N = 4 animals per group +/- SD. P values were calculated via unpaired two-sided t-test between indicated groups. **e**,**h**,**k**,**n**. Each symbol represents a tumor. Bars are the mean for each mouse +/- SD (N = 4 animals per group). P values were calculated comparing mean values between indicated groups via unpaired two-sided t-test. Source Data

The frequencies of most immune and stromal cell types were unchanged with either age or *Pten* inactivation (Extended Data Fig. 8a). However, immunohistochemical staining revealed an increase in tumor infiltration by several immune cell types with age and *Pten* deficiency (Extended Data Fig. 9a–n). Of note, the growth of PTEN-deficient tumors strongly impacted the transcriptional state of stromal and immune cells, with particularly strong effects on alveolar macrophages and endothelial cells (Extended Data Fig. 8b). These effects were also highly age-dependent: for each cell type, the majority of genes that were up- or downregulated with *Pten* inactivation were altered specifically in young or in aged cells (Fig. 4h and Extended Data Fig. 8c). Thus, aging not only shapes the consequences of PTEN inactivation on the molecular state of cancer cells, but also alters non-cell autonomous effects of the growth of PTEN-deficient tumors.

### *Pten* deficiency reduces molecular phenotypes of aging

**Extended Data Fig. 10:**
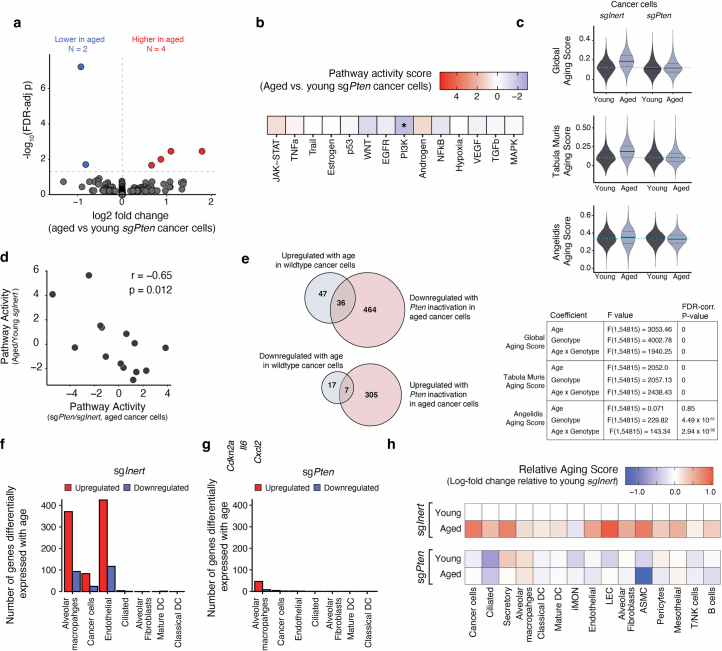
Inactivation of *Pten* decreases molecular phenotypes of aging in cancer cells. **a**. Volcano plot showing changes in gene expression with age in sg*Pten* cancer cells. Differentially expressed genes (Benjamini–Hochberg-corrected two-sided Wald test p < 0.05) are highlighted in color. **b**. Pathway activity scores comparing old to young cancer cells from sg*Pten* samples. Positive scores (red) indicate that a pathway is more active with age; negative scores (blue) indicate that a pathway is less active with age. Stars denote pathways that are differentially active with age (p < 0.05, PROGENy multivariate linear model, see Methods). Note that the color scale matches the analogous analysis in Fig. 4f. **c**. Per-cell scoring of signatures of normal aging in cancer cells (see Methods), stratified by age and genotype. Dashed blue line indicates median score for young sg*Inert* cells. Results of two-way analysis of variance shown in table below. **d**. There is a negative correlation between the effects of *Pten* inactivation in old cancer cells and the effects of aging, as measured by pathway activation. X-axis corresponds to activity scores in bottom row of Fig. 4g; Y-axis corresponds to activity scores in Fig. 4f. **e**. Venn diagrams showing overlap between genes differentially expressed with age and with *Pten* inactivation. Top: roughly 40% of genes that are upregulated with age in wild-type (sg*Inert*) cancer cells and also downregulated with *Pten* inactivation in old cancer cells. Bottom: roughly 30% of the genes that are downregulated with age in wild-type (sg*Inert*) cancer cells and also upregulated with inactivation in old cancer cells. Up/down regulation is defined by Benjamini–Hochberg-corrected two-sided Wald test p < 0.05. **f**,**g**. Number of genes differentially expressed with age in indicated cell types in mice with sg*Inert* tumors (**f**) and sg*Pten* tumors (**g**). Differential expression determined by Benjamini–Hochberg-corrected two-sided Wald test p < 0.05. Red: upregulated in old. Blue: downregulated in old. **h**. Transcriptomic ages of cells from young and old mice with sg*Inert* and sg*Pten* tumors relative to cells from young mice with sg*Inert* tumors. Cells were individually scored for Global Aging signature (see Methods). Shading reflects log fold change between the median age score for each age-genotype grouping and the median age score of young sg*Inert* cells. Source Data

To better understand the age-specific consequences of PTEN inactivation, we directly compared the transcriptomes of young and aged sg*Pten* cancer cells. Of note, young and aged sg*Pten* cancer cells had fewer differences in gene expression, muted changes in signaling pathway activation, and weaker enrichment of aging signatures relative to young and aged sg*Inert* cancer cells (Fig. 4i and Extended Data Fig. 10a,b). Likewise, per-cell aging scores of both young and aged sg*Pten* cancer cells resembled those of young sg*Inert* cancer cells, suggesting that PTEN inactivation in aged cancer cells may drive transcriptomic changes that oppose aging phenotypes (Extended Data Fig. 10c). Consistent with this, the impacts of *Pten* inactivation in aged cancer cells were negatively correlated with the effects of aging at the pathway level (*r* = −0.65), and roughly a third of the genes differentially expressed with age were regulated in the opposite direction with *Pten* inactivation in aged cancer cells (Extended Data Fig. 10d,e).

Finally, the effects of *Pten* inactivation extended to cells in the tumor microenvironment. Age-associated transcriptional changes in stromal and immune cells from mice with sg*Inert* tumors were almost entirely absent in mice with sg*Pten* tumors (Extended Data Fig. 10f–h), and most cell types in young and aged mice with sg*Pten* tumors were transcriptomically as young or younger than the corresponding cells in mice with sg*Inert* tumors (Extended Data Fig. 10h). These results suggest that *Pten* inactivation restores a youthful state in cells from aged tumors and the surrounding tissue, highlighting that the phenotypic consequences of driver mutations can both impact and be impacted by aging.

## Discussion

Understanding how aging impacts tumorigenesis is a key challenge for the field which grows more pressing as the global population ages^49,50^. Epidemiological studies have suggested a protective effect of very old age, but interpretation of these data are complicated by age-associated tumor-extrinsic factors, including differences in diagnostic intensity, patterns of environmental exposure, and the prevalence of comorbidities^11,51^. Here we used genetically engineered mouse models of human lung cancer to characterize the effects of age on tumorigenesis. By initiating tumors through the controlled induction of an oncogenic mutation, we largely uncouple tumor incidence from age-associated driver mutation accumulation, which is thought to be the dominant factor shaping cancer incidence patterns in humans. We show that aging reduces tumor initiation and growth driven by oncogenic KRAS, one of the most common oncogenic drivers across all human cancers. While reduced tumor initiation may reflect exhaustion of alveolar progenitor cells in the lung with age^52^, slower tumor growth post-initiation suggests that aging also reduces the proliferative ability of transformed cells. Our transcriptomic analysis supports this notion, as aged cancer cells fail to converge to a shared phenotypic state with young cancer cells and have reduced MAPK signaling.

We also show that aging shapes the response of KRAS-driven tumors to tumor suppressor inactivation. The impacts of several important tumor suppressor genes are dampened in aged mice, consistent with age not only reducing the average fitness of KRAS-driven lung tumors, but also constraining their maximum fitness and thereby limiting the beneficial effects of secondary mutations. This reduction in effect was especially pronounced for PTEN, a canonical negative regulator of the PI3K–AKT pathway, a central oncogenic signaling pathway in many cancer types which is activated through a variety of mechanisms in a large fraction of lung adenocarcinomas^53–55^. Our scRNA-seq analysis showed that while PTEN inactivation strongly upregulated PI3K signaling irrespective of age, the downstream phenotypic effects of this activation shifted dramatically with age in both cancer and stromal cells. These differing effects may indicate a rewiring of the signaling landscape in aged cancer cells that ultimately constrains the oncogenic potential of this important signaling axis. Surprisingly, PTEN-deficient tumors as well as co-existing stromal cells from aged mice had reduced transcriptomic signatures of aging relative to mice with PTEN-proficient tumors. This finding is unexpected given the lifespan-extending effects of reduced PI3K signaling^56–58^. Collectively, these findings underscore the interconnectedness of pathways involved in aging and tumorigenesis.

While we do not delineate the mechanisms underlying the tumor-suppressive effect of aging here, our genetic data are informative as we perturb pathways that putatively link aging and cancer. Specifically, the pro-tumorigenic effects of inactivation of p53 and Cdkn2a were not enhanced in aged mice. These data together with the lack of transcriptomic signatures of senescence suggest that senescence is unlikely to be a major contributor to the differential phenotypes in young and aged mice (Extended Data Figs. 1d and 7a–e). Likewise, inactivation of genes involved in the antitumor immune response did not strongly impact tumor fitness, suggesting that age-associated changes in adaptive immune responses and the assayed innate immune surveillance pathways are not responsible for reduced tumor initiation and growth in aged mice (Extended Data Fig. 3a–c). However, immunohistochemical staining revealed increased tumor immune infiltration with age, supporting a potential role for the aged tumor immune environment in shaping the observed phenotypes.

Given the complex and multifaceted nature of both aging and tumorigenesis, we speculate that tumor suppression with age reflects an integration of multiple mechanistic linkages between aging and cancer. Consistent with this notion, recent studies have documented both pro- and antitumorigenic effects of aging. Alterations in normal and cancer cell iron metabolism have been implicated in reducing tumor growth in an aged autochthonous lung cancer model^59^, while host age has been shown to either accelerate or have no effect on the super-physiologic growth of lung cancer cell line transplant models^60,61^. Analyses of human cancer genomic data have also documented a deleterious effect of passenger mutations accumulation, suggesting another potential mechanism that may reduce the growth of aged tumors^62,63^. Our findings that age differentially impacts the growth of tumors with different tumor suppressor alterations suggests that this integration can depend on the molecular state of tumors, underscoring the importance of further research into how age impacts the development of tumors with diverse genotypes. Future work exploring how aging impacts metastasis, response to various treatment modalities and lung cancer development from different cell types of origin could provide important insights into how age impacts the disease course and clinical outcomes of patients.

Although we focus on KRAS-driven lung adenocarcinoma in this study, a flattening and even decrease in incidence in very old age is common to most cancer types. This non-monotonic pattern could result from the competing effects of protumor driver mutation accumulation and antitumor tissue aging, where aging-induced tumor-suppressive changes begin to dominate in very old adults even as somatic cells continue to accrue genomic alterations (Supplementary Fig. 7). Additional data collected across the lifespan and in particular during ages that correspond to the peak of human cancer incidence and to extreme old age could be useful in evaluating this hypothesis and defining the interplay between the pro- and antitumorigenic effects of aging. Notably, both the dynamics of mutation accumulation and the tumor-repressive effects of aging are likely tissue-dependent, necessitating further investigation on the impacts of aging in different cancer types and tissue contexts. A key future challenge will be to identify the specific aspects of aging that suppress tumor growth so that they can be harnessed separately from the protumorigenic and pathological effects of aging.

## Methods

### Mice, tumor initiation and tissue collection

The use of mice for this study was approved by the Institutional Animal Care and Use Committee at Stanford University, protocol number 26696. *Kras*^*LSL-G12D/+*^ (RRID:IMSR_JAX:008179), *p53*^*flox/flox*^ (RRID:IMSR_JAX008462), *R26*^*LSL-tdTomato*^ (RRID:IMSR_JAX:007914) and *H11*^*LSL-Cas9*^ (RRID:IMSR_JAX:027632) mice have been previously described^20,21,64,65^. All mice were on a *C57BL/6* background. The sex of animals was balanced in each cohort in each experiment. Ages of mice used in each experiment are indicated in the figures. Mice were housed in 12-h light–dark cycles at room temperature ranging between 20 °C and 26 °C and humidity between 30% and 70%. Mice were fed ad libitum with 2018 Teklad 18% protein rodent diet (Envigo) and provided with ad libitum water. Mice housed in Innovive caging received Aquavive Mouse Pre-filled Acidified Water Bottles (Innovive).

Lung tumors were initiated by intratracheal intubation and delivery of 60 μl of lentiviral vectors in PBS to isoflurane-anesthetized mice. Lentiviral titers and durations of tumor growth are indicated in the figures.

Lungs were weighed at the time of collection and cancer cell weight (tumor-bearing lung weight minus normal lung weight) was calculated as a metric of overall tumor burden. Tumor burden was also assessed via fluorescence imaging and quantified using ImageJ. Lung lobes were frozen for DNA extraction and Tuba-seq analysis, fixed for histology, and/or dissociated into a single-cell suspension for molecular analyses.

Young and aged mice used in analyzing the impact of age on the efficiency of lentiviral transduction of lung epithelial cells (Fig. 1e and Supplementary Fig. 2) were *C57BL/6* mice obtained from the National Institute of Aging’s aged rodent colony. These mice were housed in the Stanford SIM1 barrier facility for at least 2 months before experimentation.

### Design and generation of Lenti-sgRNA/Cre vectors

Most barcoded Lenti-sgRNA/Cre vectors have been previously described^19^. New sgRNA sequences targeting *Pten* were designed using CRISPick (https://portals.broadinstitute.org/gppx/crispick/public). sgRNA sequences are presented in Supplementary Table 1. Each desired sgRNA vector was modified from pll3-U6-sgRNA-Pgk-Cre vector via site-directed mutagenesis (New England Biolabs, E0554S). The generation of the barcode (BC) fragment containing the eight-nucleotide sgID sequence and 20-nucleotide degenerate BC, and subsequent ligation into the vectors, were performed as previously described^18,19^. The Lenti-sgRNA^Immune^/Cre pool consisted of ‘Tuba-seq^Ultra^’ vectors with clonal BC sequences encoded within the 20-nucleotide region at the 3’ end of the U6 promoter directly adjacent to each sgRNA. These vectors were generated as previously described^66^.

Lenti-sgRNA/Cre vectors were individually co-transfected into 293T cells with pCMV-VSV-G (Addgene, #8454) envelope plasmid and pCMV-dR8.2 dvpr (Addgene, #8455) packaging plasmid using polyethyleneimine in 150-mm cell culture plates. Sodium butyrate (Sigma-Aldrich, B5887) was added 8 h after transfection to achieve a final concentration of 20 mM. Medium was refreshed 24 h after transfection. Supernatants were collected at 36 and 48 h after transfection, filtered through a 0.45-μm syringe filter (Millipore, SLHP033RB) to remove cells and debris, concentrated by ultracentrifugation (25,000*g* for 1.5 h at 4 °C), and resuspended overnight in PBS then frozen at −80 °C.

### Lentiviral titering and pooling

Concentrated lentiviral particles were titered by transducing LSL-YFP cells (a gift from A. Sweet-Cordero/UCSF), determining the percent YFP-positive cells by flow cytometry, and comparing the titer to a lentiviral preparation of a known titer. Vectors for the Tuba-seq studies in *K;H11*^*LSL-Cas9*^ and *KP;H11*^*LSL-Cas9*^ mice were pooled with the goal of producing roughly equal tumor burden per vector in the young mice based on previous Tuba-seq data. Specifically, all vectors were pooled at equal titers with the exception of vectors targeting genes known to be strong tumor suppressors (*Pten*, *Lkb1*, *Nf1*, *Stag2* and *Setd2*), which were pooled at half titers. For calculations of tumor-suppressive effect, the exact representation of each vector in the viral pool was determined through analysis of tumor-bearing lungs from Cas9-negative *K* control mice (see ‘Adaptive sampling of tumors for statistical comparison of tumor genotypes’).

### Immunohistochemistry

Tissues were fixed in 4% formalin for 24 h, stored in 70% ethanol and paraffin-embedded. Hematoxylin and eosin (H&E) staining was performed on 4-µm thick sections by Histo-Tec Laboratory using standard protocols. Tumors were graded by a pathologist as previously described^24^.

Antigen retrieval for HMGA2, NKX2.1 and GFP was performed with 10 mM citrate buffer in a pressurized decloaking chamber. For HMGA2 and NKX2.1, slides were washed with 1× PBST and prepared with the VECTASTAIN ABC-HRP Kit (Vector Laboratories, PK-4000). Slides were incubated overnight at 4 °C with 1:250 dilution of NKX2.1 antibody (Abcam, ab76013) or 1:500 dilution of HMGA2 antibody (Biocheck, 59170AP). Slides also stained for GFP were washed with 1× TBST before preparation with the VECTASTAIN ABC-AP kit (Vector Laboratories, AK-5001) and overnight incubation at 4 °C with 1:100 dilution of GFP antibody (Cell Signaling Technology, mAb 2956). Sections were developed with DAB (Vector Laboratories, SK-4100) or Vector Red (Vector Laboratories, SK-5100) and counterstained with hematoxylin.

Immunohistochemistry for CD45, B220, CD4 and CD8 was performed on paraffin-embedded sections using a Leica BOND RXm Automatic Slide Stainer with either a Leica BOND Polymer Refine Detection kit (Leica Biosystems, DS9800) or a Leica BOND Intense R Detection kit (Leica Biosystems, DS9263) for rabbit or rat antibodies, respectively. After deparaffinization and citrate heat-induced antigen retrieval, sections were incubated with the following antibodies for 30 min at room temperature: CD45 (1:200 dilution, Cell Signaling, 70257S, clone D3F8Q), B220 (1:1,500 dilution, BD Biosciences, 550286, clone RA3-6B2), CD4 (1:200 dilution, Cell Signaling, 25229S, clone D7D2Z) and CD8 (1:200 dilution, Cell Signaling, 98941S, clone D4W2Z). Sections were then incubated with a poly-HRP anti-rabbit IgG polymer (Leica Biosystems, BOND Polymer Refine Detection kit, DS9800) or an anti-rat IgG ABC reagent (Vector Laboratories, PK-6104) for 8 min at room temperature. Color precipitate was developed with DAB chromogenic substrate (Leica Biosystems, BOND Polymer Refine Detection kit, DS9800; Leica Biosystems, BOND Intense R Detection kit, DS9263) followed by counterstaining with hematoxylin (Leica Biosystems, BOND Polymer Refine Detection kit, DS9800; Leica Biosystems, BOND Intense R Detection kit, DS9263). Whole-slide scans were generated using a Leica Aperio VERSA Slide Scanner Microscope at ×10 magnification. Using these scans, staining was quantified by calculating the number of positive cells within each tumor via the publicly available software QuPath (v.0.5.1; https://qupath.github.io/). Representative photomicrographs of staining were captured on a Leica DMI6000B inverted light and fluorescence microscope at ×20 magnification.

### Transduction efficiency analysis

To assess the impact of age on lentiviral transduction efficiency of lung epithelial cells, young and aged mice were intratracheally transduced with 1.0 × 10^6^ infectious units of a *GFP*-expressing lentiviral vector or with an equivalent volume of PBS (untransduced controls). To allow time for expression of GFP in transduced cells, lungs were collected 7–8 days after transduction. Lungs were inflated with digestion medium containing collagenase IV, dispase and trypsin and dissociated at 37 °C for 30 min. Cells were stained with 4,6-diamidino-2-phenylindole (DAPI) and antibodies against lineage markers CD45 (30-F11), CD31 (390), F4/80 (BM8), Ter119 and epithelial marker EpCAM (all from BioLegend and diluted 1:800). Transduction efficiency was measured by fluorescence-activated cell sorting (FACS) for each sample via quantification of the percentage of live epithelial cells (EpCam^pos^Lineage^neg^DAPI^neg^) in each sample that was GFP-positive. Untransduced control samples were used to define an appropriate gating strategy.

### Tuba-seq library generation and sequencing

For Tuba-seq library generation, genomic DNA was isolated from whole tumor-bearing lung tissue from each mouse as previously described^67^. Three benchmark control ‘spike-in’ cell lines (10^5^ cells each) were added to each lung sample before lysis to enable the calculation of the absolute number of neoplastic cells in each tumor from the number of sgID-BC reads^18^. Following homogenization and overnight protease K digestion, genomic DNA was extracted from the lung lysates using phenol–chloroform and ethanol precipitation. Libraries were prepared by amplifying the sgID-BC region from 32 μg of genomic DNA per mouse using unique dual-indexed primers and the Q5 Ultra II 2× Master Mix (New England Biolabs, M0544X). The PCR products were purified with Agencourt AMPure XP beads (Beckman Coulter, A63881). The concentration and quality of the purified libraries were determined using the Tapestation (Agilent Technologies). Tuba-seq^Ultra^ libraries for mice transduced with the Lenti-sgRNA^Immune^/Cre pool were generated as previously described^66^.

Unequal sequencing depth can be a source of noise in characterizing the distribution of tumor sizes across samples, and a potential confounding factor if systematically different across experimental groups. To ensure that samples were sequenced evenly, we performed two sequential rounds of paired-end sequencing for each set of Tuba-seq samples. Sequencing depths were ascertained using the read counts of the spike-in cell lines in the first round of sequencing and were then used to adjust sample pooling for the second round to ensure equal overall sequencing depth. For both experiments using Lenti-sg*RNA*^*Aging*^/*Cre*, samples were divided into two sub-pools and sequenced on the Illumina MiSeq Nano, re-pooled, and then sequenced a second time on the Illumina NextSeq 500 platform (read lengths 2 × 150 bp, through Admera Health). To minimize the influence of batch effects associated with each sequencing lane, sub-pools were balanced for the sex and age of mice. For the individual gene validation experiments, samples were sequenced on a partial lane of Illumina NovaSeq 6000, re-pooled, and then sequenced on a second partial lane of NovaSeq 6000 (2 × 150 bp, through Novogene) or were pooled on the basis of lung weight (a proxy for tumor burden). Three of the samples transduced with Lenti-sg*RNA*^*Aging*^/*Cre* had extremely aberrant sequencing depths and were removed from the analysis due to assumed failures in library preparation (an aged *Kras*^*LSL-G12D/+*^*;H11*^*LSL-Cas9*^ mouse, an aged *Kras*^*LSL-G12D/+*^*;p53*^*flox/flox*^*;H11*^*LSL-Cas9*^ mouse and a *Kras*^*LSL-G12D/+*^ mouse).

### Analysis of sgID-BC sequencing data

Sequencing of Tuba-seq libraries produces reads that are expected to contain an eight-nucleotide sgID followed by a 30-nucleotide BC of the form GCNNNNNTANNNNNGCNNNNNTANNNNNGC, where each of the 20 Ns represents a random nucleotide. Each sgID has a one-to-one correspondence with an sgRNA in the viral pool (Supplementary Table 1); thus, the sgID sequence identifies the gene targeted in each tumor. Note that all sgID sequences in a viral pool differ from each other by at least three nucleotides such that incorrect sgID assignment (and thus, inference of tumor genotype) due to PCR or sequencing error is extremely unlikely. The random 20-nucleotide BC tags all cells in a single clonal expansion. Note that the length of the BC ensures a high theoretical potential diversity, with the actual diversity of each Lenti-sgRNA/Cre vector dictated by the number of bacterial colonies pooled during the plasmid barcoding step.

FASTQ files were parsed using regular expressions to identify the sgID and BC in each read. To minimize the effects of sequencing error on BC identification, we required the forward and reverse reads to agree completely within the sgID-BC region^35^. We also performed an analysis to identify BCs that were likely to have arisen from genuine tumors due to PCR or sequencing errors. Given the low rate of sequencing error, we expect these ‘spurious tumors’ to have read counts that are far lower than the read counts of the genuine tumors from which they arise. To minimize the impact of these ‘spurious tumors’, we identified small ‘tumors’ with BCs that were highly similar to the BCs of larger tumors in the same sample. Specifically, if a pair of ‘tumors’ within a sample had BCs that were within a Hamming distance of two, and if one of the tumors had fewer than 5% as many reads as the other, then the reads associated with the smaller tumor were attributed to the larger tumor.

After these filtering steps, the read counts associated with each BC were converted to absolute neoplastic cell numbers by normalizing to the number of reads from the ‘spike-in’ cell lines added to each sample before lung lysis and DNA extraction.

Tuba-seq^Ultra^ libraries were analyzed using a modified pipeline as previously described^66^.

### Removal of contaminating barcodes

sgID-BC sequences that are not from genuine tumors in an individual tumor-bearing lung sample can, nonetheless, be present in sequencing libraries for several reasons, including intra-experiment sample-to-sample cross-contamination during library preparation, external contamination (for example from samples and libraries from other experiments) and library-to-library misassignment during sequencing. These sgID-BC sequences have the potential to be identified as small tumors (‘spurious tumors’) and thereby reduce the precision of our analyses. Both external contamination and sample-to-sample contamination result in the identification of the same sgID-BC in multiple samples. However, some sgID-BCs are expected to recur across samples in the absence of contamination due to the finite diversity of the sgID-BC region in each Lenti-sgRNA/Cre vector. To minimize the effects of contamination, we examined patterns of sgID-BC recurrence across samples and removed barcodes that occurred in a number of samples that would be highly unlikely to occur by chance given the BC diversity of each vector.

To estimate the BC diversity associated with each sgID, we assume that the probability of observing a BC in *i* mice is Poisson distributed: P(*k* = *i*; λ) = *λ*^k^ *e*^−*λ*^ */ k*!, where *λ* is the mean number of mice that barcodes appear in for a given Lenti-sgRNA vector. To estimate *λ*_*r*_ for each sgID *r* in the dataset we note that *λ*_*r*_/(1 – *e*^*−λ*r^) = *μ*_non-zero_, where *μ*_non-zero_ = Σ_*i*=1_^∞^P(*k* = *i*; *λ*_*r*_) is the mean number of occurrences of each BC that occurred once or more (a known quantity). Given the Poisson distribution defined for each sgID *r*, we calculated a Lenti-sgRNA/Cre vector-specific threshold *N*_*r*_ such that 99.9% of barcodes would be expected to appear in *N*_*r*_ samples or fewer and identified and removed all ‘tumors’ with sgID-BCs that occurred in a number of samples exceeding *N*_*r*_.

sgID-BCs can also recur across samples due to misassignment of reads during sample de-multiplexing. While misassignment of reads is expected to be extremely rare, it could result in sgID-BCs from genuine tumors with very large read counts in one sample (a very large, genuine tumor) appearing with low read counts in additional samples sequenced in the same lane. To guard against this possibility and ensure that very large tumors were not systematically discarded by the procedure described in the previous paragraph, we examined the distribution of reads across samples to identify sgID-BCs that appeared in >*N*_*r*_ samples but where >95% of sequencing reads were assigned to a single sample. These sgID-BCs were retained in the sample with the bulk of the sequencing reads and discarded in all other samples. All other barcodes appearing in >*N*_*r*_ samples were discarded from all samples.

### Removal of mice with aberrantly few barcodes

After processing sequencing data, the number of unique barcodes was tallied in each sample. The barcode number across mice was highly variable for both young and aged mice, which likely reflects variability in the success of lentiviral transduction due to technical reasons. Samples with an extremely low barcode number (fewer than 1,000 barcodes, compared to an average of 87,868 barcodes detected in mice transduced with Lenti-sg*RNA*^*Aging*^/*Cre*) were deemed to have not been successfully transduced and were removed from the analysis. On this basis, we removed one young and one aged sample from the cohort of *Kras*^*LSL-G12D/+*^*;H11*^*LSL-Cas9*^ mice transduced with Lenti-sg*RNA*^*Aging*^/*Cre* and two young samples from the cohort of *Kras*^*LSL-G12D/+*^*;p53*^*flox/flox*^*;H11*^*LSL-Cas9*^ mice transduced with Lenti-sg*RNA*^*Aging*^/*Cre*.

### Filtering of vectors with insufficient titer from analysis

Although Lenti-sgRNA/Cre vectors were titered before pooling to aim for the desired representation in the Lenti-sg*RNA*^*Aging*^/*Cre* viral pool, the distribution of vectors in the viral pool was uneven. Examination of the number of tumors associated with each sgRNA in the *K;H11*^*LSL-Cas9*^ mice revealed that a small number of vectors had titers roughly an order of magnitude lower than the median titer (<3,000 unique barcodes relative to the median of 46,793). Vectors with fewer than 3,000 unique barcodes identified across the young and aged *K;H11*^*LSL-Cas9*^ mice were deemed to be insufficiently represented and were filtered out before analysis. Critically, all filtered vectors were similarly poorly represented in the *Kras*^*LSL-G12D/+*^ control mice (<750 unique barcodes per vector, relative to the median of 9,695), indicating that the low barcode counts in the experimental cohorts were due to uneven pooling rather than the impacts of gene targeting.

### Adaptive sampling of tumors for statistical comparison of tumor genotypes

In comparing the effects of tumor suppressor inactivation in young and aged mice, we sought to compare equivalent portions of the tumor size distributions (the same number of tumors per infectious unit of virus delivered) for each sgRNA in the young and aged cohort. To ensure this, we scaled the number of tumors analyzed for each sgRNA *i* in each cohort *j* to account for differences in viral titer and the number of mice transduced in each cohort, and then analyzed the largest *N*_*i*__,__*j*_ tumors per Lenti-sgRNA/Cre vector.

This scaling procedure requires selecting a benchmark sgRNA and a benchmark cohort, and then selecting a defined number of tumors with that benchmark sgRNA from that cohort. For each analysis, we used sg*NT1* in the young cohort as the benchmark dataset. Note that this choice was arbitrary, and that identical results could be achieved by basing the sampling calculations on any sgRNA in either the young or aged cohort. The number of tumors sampled for each other sgRNA *i* in each cohort *j* (*N*_*i*,*j*_) is then adjusted to take into account the proportions of sgRNAs in the viral pool and differences in the overall viral titer delivered to the young and aged cohorts:

$${N}_{i,j}={N}_{{i}={\mathit{NT1}},{j}={\mathrm{Young}}}\times \frac{{T}_{j}}{{T}_{{j}={\mathrm{Young}}}}\times \frac{{p}_{i,j}}{{p}_{{i}={\mathit{NT1}},{j}={\mathrm{Young}}}}$$

where *T*_*j*_ denotes the total viral titer delivered to cohort *j* and *p*_*i*,*j*_ denotes the proportion of the viral pool in delivered to cohort *j* that is allocated to sgRNA *i*. *T*_*j*_ is a known quantity determined during viral preparation which represents the sum of the viral titers delivered to each mouse in cohort *j*. *p*_*i*,*j*_ values were calculated using Tuba-seq data generated by transducing control *Kras*^*LSL-G12D/+*^ mice. As these mice lack Cas9 expression, all Lenti-sgRNA/Cre vectors are functionally inert, and the observed tumor number associated with each sgRNA reflects the make-up of the viral pool. To account for variation in tumor number across mice the sample of *N*_*i*,*j*_ tumors was distributed across all mice in the cohort in proportion to the total number of tumors in each mouse.

### Selection of number of tumors to sample in benchmark dataset (*N*_*i*=*NT1*,*j*=Young_)

The parameter *N*_*i*=*NT1*,*j*=Young_ defines the portion of the tumor size distribution that we use in assessing the impact of age on tumorigenesis and tumor suppressor function. In selecting *N*_*i*=*NT1*,*j*=Young_ for each analysis we sought to include as much data as possible while maintaining high data quality by excluding tumors with barcodes supported by very few reads. We reasoned that restricting our analysis to tumors supported by an average of >5 reads in each dataset would minimize noise associated with the detection and measurement of very small tumors, while maintaining high statistical power to detect the impacts of aging on tumor-suppressive effects. We therefore selected an *N*_*i*=*NT1*,*j*=Young_ for each analysis that corresponded to the number of sg*NT1* tumors supported by at least five reads in the young cohort (rounded to the nearest 100). These values of *N*_*i*=*NT1*,*j*=Young_ are shown in the table below. To ensure that our findings were robust to variation in the chosen values of *N*_*i*=*NT1*,*j*=Young_, we performed sensitivity analyses by increasing/decreasing *N*_*i*=*NT1*,*j*=Young_ by 10%, 25% and 50% and found similar results (Extended Data Fig. 2).

**Table:** 

Cohort description (virus, mice)	Number of tumors sampled in benchmark dataset (*N*_*i*=*NT1*,*j*=Young_)	Corresponding figures
Lenti-sg*RNA*^*Aging*^/*Cre* (all *K;H11*^*LSL-Cas9*^)	4,600	Fig. 2 and Extended Data Figs. 1 and 2
Lenti-sg*RNA*^*Aging*^/*Cre* (all *KP;H11*^*LSL-Cas9*^)	1,800	Fig. 3 and Extended Data Figs. 2 and 3
Lenti-sg*RNA*^*Aging*^/*Cre* (all *K*)	2,100	Extended Data Fig. 1
Lenti-sg*RNA*^*Aging*^/*Cre* (male *K;H11*^*LSL-Cas9*^)	3,100	Supplementary Fig. 5
Lenti-sg*RNA*^*Aging*^/*Cre* (female *K;H11*^*LSL-Cas9*^)	1,500	Supplementary Fig. 5
Lenti-sg*RNA*^*Aging*^/*Cre* (male *KP;H11*^*LSL-Cas9*^)	1,100	Supplementary Fig. 5
Lenti-sg*RNA*^*Aging*^/*Cre* (female *KP;H11*^*LSL-Cas9*^)	700	Supplementary Fig. 5
Lenti-sg*Safe*^*Pool*^/*Cre* (all *K;H11*^*LSL-Cas9*^)	88,000	Extended Data Fig. 1
Lenti-sg*RNA*^*Immune*^/*Cre* (all *K;H11*^*LSL-Cas9*^)	2,600	Extended Data Fig. 3

### Summary statistics for impact of gene inactivation on tumorigenesis

After performing the adaptive sampling described above, we assessed the extent to which each targeted gene (*X)* affects tumor initiation and growth by comparing the distribution of tumor sizes produced by vectors targeting that gene (sg*X* tumors) to the distribution produced by our negative control vectors (sg*Inert* tumors). We characterized these distributions using the maximum likelihood estimate of mean tumor size assuming a log-normal distribution (LN mean). Previous work found that this statistic represents the best parametric summary of tumor growth based on the maximum likelihood quality of fit of various common parametric distributions^18^. In addition, we calculated the sizes of tumors at various percentiles of the distribution (50th, 60th, 70th, 80th and 90th) for each sgRNA as complementary nonparametric summary statistics.

Note that while these statistics are functions of tumor size distribution, they are also sensitive to the effects of tumor suppressor gene inactivation on tumor initiation due to our use of adaptive sampling. For example, a gene that increases tumor burden when inactivated solely by increasing the number of tumors (without shifting the tumor size distribution) would still increase the LN mean of the adaptively sampled datasets and would therefore still be detectable as a tumor suppressor using these metrics. These statistics therefore integrate the impact of each targeted gene on the initiation and growth of tumors.

To quantify the extent to which each gene suppressed or promoted tumorigenesis, we normalized statistics calculated on tumors of each genotype to the corresponding sg*Inert* statistic. The resulting ratios reflect the fitness advantage (or disadvantage) associated with each tumor genotype relative to the initiation and growth of *sgInert* tumors.

The adaptively sampled relative LN mean size for tumors of genotype X was calculated as:

$$\mathrm{Relative}\,\mathrm{LN}\,\mathrm{mean}\,{\mathrm{size}}_{\mathrm{sg}X}=\frac{\mathrm{LN}\,\mathrm{mean}\,\mathrm{of}\,\mathrm{sg}X\,\mathrm{distribution}}{\mathrm{LN}\,\mathrm{mean}\,\mathrm{of}\,\mathrm{sg}{Inert}\,\mathrm{distribution}}$$

Likewise, the adaptively sampled relative *i*th percentile for tumors of genotype X was calculated as:

$${\mathrm{Relative}\,i\mathrm{th}\,\mathrm{percentile}\,\mathrm{size}}_{\mathrm{sg}X}=\frac{i\mathrm{th}\,\mathrm{percentile}\,\mathrm{of}\,\mathrm{sg}X\,\mathrm{distribution}}{i\mathrm{th}\,\mathrm{percentile}\,\mathrm{of}\,\mathrm{sg}{Inert}\,\mathrm{distribution}}$$

where the sg*X* and sg*Inert* distributions were adaptively sampled as previously described and the sg*Inert* statistic is the median value across all inert vectors.

### Calculation and interpretation of scoreRGM

To corroborate our adaptively sampled comparison of tumor suppressor effects in young and aged mice, we used a pre-existing method designed to compare tumor-suppressive effects across cohorts of mice treated with different therapies (‘scoreRGM’)^35^. Like the ASM, scoreRGM seeks to ensure that equivalent portions of the tumor size distribution are analyzed for each sgRNA in each context. To do so, this method leverages the number and size of sg*Inert* tumors in young and aged mice to explicitly account for changes in tumor growth dynamics with age that are independent of tumor suppressor genotype, and then infers age-by-tumor-suppressor interactions as deviations from this baseline.

Specifically, for a given minimum tumor size cutoff *L* applied in the aged mice (in Extended Data Fig. 2; 500 cells), we calculate a reduction factor *R*, which is the proportional reduction in tumor size that when applied to the young mice minimizes the difference between the median number of sg*Inert* tumors in young and aged mice. *R* can be used to define an adjusted cutoff *L* × *R*, which we would expect to capture the equivalent portion of the tumor size distribution in the young mice. To contrast the effects of an sgRNA targeting gene *X* we then calculate:

$${\mathrm{scoreRGM}}_{X}={\log }_{2}\left(\frac{{\mathrm{GM}}\left[{\mathrm{sg}}{X}_{{\mathrm{aged}},{p}_{X}\times {N}_{L}}\right]/{\mathrm{GM}}[{\mathrm{sg}}{\it{Inert}}_{{\mathrm{aged}},{N}_{L}}]}{{\mathrm{GM}}\left[{\mathrm{sg}}{X}_{{\mathrm{young}},{{p}_{X}\times N}_{L\times R}}\right]/{\mathrm{GM}}\left[{\mathrm{sg}}{\it{Inert}}_{{\mathrm{young}},{N}_{L\times R}}\right]}\right)$$

where ‘GM’ denotes the geometric mean calculated over the bracketed set of tumors, *N*_*L*_ is the number of sg*Inert* tumors larger than *L* in the aged mice, *N*_*L*×*R*_ is the equivalent number of sg*Inert* tumors in the young mice and *p*_*X*_ is the ratio of tumors with sg*X* to sg*Inert* tumors. Thus, scaling the number of tumors analyzed by *R* adjusts for the impact of age on the tumor size distribution, and scaling by *p*_*X*_ ensures that an equivalent number of tumors are analyzed for each sgRNA.

Under the null hypothesis that gene *X* has the same effect in young and aged mice, the ratio of the geometric means of sg*X* and sg*Inerts* in aged mice (numerator) will be equivalent to the ratio of geometric means in the young mice (denominator) and scoreRGM_*X*_ = 0. A positive value of scoreRGM_*X*_ indicates that gene *X* is a stronger tumor suppressor in aged mice than in young mice; a negative value of scoreRGM_*X*_ indicates that gene *X* is a stronger tumor suppressor in young mice than in aged mice.

### Aggregating information across sgRNAs in calculation of tumor growth metrics

Adaptive sampling and calculation of summary statistics were performed per sgRNA. To calculate statistics at the gene level, we aggregated information across all sgRNAs targeting the same gene. Specifically, the gene-level statistics reported are the weighted average of the per sgRNA statistics, where the contribution of each sgRNA was proportional to the number of adaptively sampled tumors with that sgRNA.

### Calculation of confidence intervals and *P* values for impacts of gene inactivation on tumorigenesis

Confidence intervals and *P* values were calculated using bootstrap resampling to estimate the sampling distribution of each statistic. To account for both mouse-to-mouse variability and variability in tumor size and number within mice, we used a two-step, nested bootstrap approach where we first resampled mice, and then resampled tumors within each mouse in the pseudo-dataset. The 10,000 bootstrap samples were drawn for all reported *P* values. Then, 95% confidence intervals were calculated using the 2.5th and 97.5th percentiles of the bootstrapped statistics. Because we calculate metrics of tumor growth/burden that are normalized to the same metrics in sg*Inert* tumors, under the null model where genotype does not affect tumor growth the test statistic is equal to 1. Two-sided *P* values were calculated as follows:

$$P=2\times \min \{\Pr \left(T > 1\right),\Pr \left(T < 1\right)\}$$

where *T* is the test statistic and Pr(*T* > 1) and Pr(*T* < 1) were calculated empirically as the proportion of bootstrapped statistics that were more extreme than the baseline of 1. To account for multiple hypothesis testing, *P* values were false discovery rate (FDR)-adjusted using the Benjamini–Hochberg procedure as implemented in the Python package stats models or in R Stats package.

### Calculation of *P* values for differential effects of tumor suppressor inactivation with age

To compare the impacts of inactivating a given tumor suppressor gene in young and aged mice, we defined a test statistic *T* equal to the difference between the effect in young and aged. We produced a bootstrapped distribution of *T* by resampling mice and tumors within each cohort (as described above) and repeatedly calculating *T*. Under the null hypothesis, the impact of inactivating a given tumor suppressor gene is the same in young and aged and *T* is therefore equal to 0. Two-sided *P* values were calculated as follows:

$$P=2\times \min \{\Pr \left(T > 0\right),\Pr \left(T < 0\right)\}$$

where Pr(*T* > 0) and Pr(*T* < 0) were calculated empirically as the proportion of bootstrapped statistics that were more extreme than the baseline of 0. To account for multiple hypothesis testing, *P* values were FDR-adjusted using the Benjamini–Hochberg procedure as implemented in the Python package stats models or in the R Stats package.

### Comparison of tumor size distributions

In addition to quantifying the impact of each gene on tumorigenesis in each age context, we also leveraged the barcode sequencing data to compare the overall distributions of tumor sizes between young and aged mice. To visualize differences in the tumor size distribution between groups of mice we plotted the empirical cumulative distribution function (CDF) for each group using the R function stat_ecdf. CDFs describe the probability that a random variable takes a value that is less than or equal to a given number- in this case, the percent of tumors that are as small or smaller than a given size. To ensure that equivalent portions of the tumor size distribution factored into each comparison, the number of tumors included in each group was scaled to account for differences in viral titer. Tumor size distributions were compared statistically using the K–S test.

### Preparation of bulk-tumor-bearing lungs for scRNA-seq

Whole tumor-bearing lungs were finely minced immediately after collection and enzymatically dissociated using collagenase IV, dispase and trypsin at 37 °C for 30 min as previously described^68^. Bulk cell suspensions were viably frozen in Bambanker cryopreservation medium (Wako Chemicals, 302-14681) at −80 °C before transferring to liquid nitrogen. Cells were thawed and stained with antibodies against CD45 (30-F11), CD31 (390), F4/80 (BM8) and Ter119 (all from BioLegend and diluted 1:800) to identify hematopoietic and endothelial cells. Each sample was sorted for viability (DAPI-negative) and divided into two compartments: (1) ‘cancer cell’ samples containing Tomato^pos^, CD45^neg^, CD3^neg^, F4/80^neg^ and Ter119^neg^ cells which are presumed to be cancer cells, and (2) ‘bulk’ samples containing all other viable cells. FACSAria sorters (BD Biosciences) were used for all cell sorting. Samples were fixed immediately after sorting using Parse Biosciences Cell Fixation kits (v.2, May 2023) and stored at −20 °C. To maximize recovery for cancer cell samples where we had limited input material, we used a modified version of the Parse fixation protocol. Specifically, all volumes were scaled down to one-quarter of the recommended amount so that the entirety of the final suspension of fixed cells could be loaded into the plate used for barcoding and library preparation.

### Single-cell RNA sequencing

Fixed cell suspensions were thawed and visually inspected under the microscope for the presence of debris, and one bulk sample was excluded on this basis (from mouse MT1906). The remaining 31 samples were processed for multiplexed scRNA-seq using the Parse Biosciences WT Mega v.2 kit according to the manufacturer’s instructions. This kit uses a combinatorial barcoding approach, allowing multiplexing of all samples into 16 sub-libraries, each containing a mixture of cells from all samples. Samples for this experiment were loaded into the kit and processed alongside and sequenced with samples from another experiment. Sub-libraries were quantified by Qubit fluorometer, pooled to equalize the amount of DNA per cell, and sequenced on an Illumina NovaSeq S4 flow cell to an average depth of 25,901 reads per cell.

### scRNA-seq data processing and quality control

The processing pipeline provided by Parse Biosciences (split-pipe v.1.1.1) was used with default settings to align sequencing reads to the GRCm38 mouse reference genome. Each of the 16 sub-libraries was first processed individually using the command split-pipe –mode all, and the output of the 16 sub-libraries was combined using split-pipe –mode combine.

Cell barcodes were filtered to include only cells meeting the following criteria: (1) expressing between 200 and 10,000 genes; (2) having between 500 and 100,000 read counts; and (3) having less than 10% of reads map to mitochondrial genes. Scrublet (v.0.2.3) was used to simulate doublets and assign doublet scores to cells based on a *k*-nearest-neighbor classifier^69^. Cells with doublet scores >0.12 were removed from the dataset on the basis of visual inspection of the doublet score density plot for simulated doublets and inspection of uniform manifold approximation and projection (UMAP) embeddings. Read counts were normalized per cell using the scanpy (v.1.9.8) function ‘normalize_total’ with default parameters and scaled for variance stabilization using a shifted logarithm transformation (log *X* + 1, where *X* is the normalized counts).

### Dimensionality reduction, clustering and visualization of single-cell profiles

After filtering and normalization, feature selection was performed on the logarithmized data to identify transcriptionally over-dispersed genes. Specifically, the scanpy function ‘highly_variable_genes’ was used to bin genes based on their mean expression and then annotate genes with high levels of dispersion relative to genes in the same bin. UMAP embedding was then performed to visualize single-cell profiles in two-dimensional space^70^. Leiden clustering was performed at various resolutions to aid in cell type assignment (see below)^71^.

### Cell type assignment

Cell types were assigned after quality control and doublet removal on the basis of marker gene expression using Semi-supervised Category Identification and Assignment (SCINA^36^, v.1.2.0). Cell-type identities and corresponding marker genes were taken from the LungMAP Consortium’s Mouse cell reference, accessed through the LGEA Web Portal (https://research.cchmc.org/pbge/lunggens/CellRef/LungMapCellRef.html)^72,73^. For each cell type, all genes listed as positive signatures in LungMAP were used as marker genes. SCINA was run using default model parameters.

SCINA assigns cell types to each cell individually. To assign types to cells that were not successfully annotated (annotated with type ‘unknown’) and ensure homogenous cell type assignments within clusters, we refined this initial clustering through two rounds of cluster-based annotation. In the first round, a coarse-grained clustering was performed to separate cells by broad lineage identity (epithelial, mesenchymal, endothelial, myeloid and lymphoid). Specifically, cells were clustered using the Leiden algorithm implemented in scanpy with a resolution parameter of 0.5 (ref. ^71^). Each cluster was assigned a lineage identity based on the majority lineage identity of its constituent cells (where the mapping of cell type to lineage identity was specified by LungMAP cell reference). The dataset was then subdivided into epithelial cells, mesenchymal cells, endothelial cells, myeloid cells and lymphoid cells. A finer-grained clustering with a resolution parameter of 1.0 was then performed within each these broad cell types. Final cell types were then assigned on a per-cluster basis based on the majority cell type of the constituent cells in each cluster.

After automatic annotation, cell type annotations were manually reviewed. Clusters with membership equally spread across two or more cell types were annotated with a more generic cell type label encompassing the component cell types. Specifically, clusters containing mixtures of T regulatory, CD4^+^ T cells, CD8^+^ T cells and natural killer (NK) cells were annotated as T lymphocytes/NK cells; clusters containing mixtures of alveolar fibroblast 1 and 2 cells were annotated as alveolar fibroblasts; and mixtures of endothelial cells and capillary cells were annotated as endothelial cells. In the rare instances where this was not possible, clusters were annotated as ‘unknown’ and removed from downstream analysis.

### Removal of transduced non-cancer cells

Although lentivirus primarily has tropism for alveolar epithelial cells, we expect rare transduction of other cell types^74^. Although these events are not expected to give rise to tumors, they could impact the molecular state of the transduced cells. To minimize these effects, we removed all non-epithelial cells that either expressed *tdTomato* transcripts or were sorted into cancer cell samples during our sample preparation for scRNA- seq (and therefore have a higher likelihood of expressed Tomato at the time of sorting).

### Definition of cancer cell population

Lung adenocarcinomas arise from cells in the alveolar epithelium^75,76^. Consistent with this, *Tomato* expression in our dataset mapped primarily to AT1-, AT2- and AT/AT1-like cells. It is, however, likely that a portion of these alveolar cells were not transduced. While in theory *Tomato* expression serves as a marker of transduction, expression of the transgene was insufficiently high to serve as a reliable marker of cancer cell identity (exclusion on the basis of failure to express *Tomato* would discard a large number of likely cancer cells) (Supplementary Fig. 6). We reasoned that AT1, AT2 and AT1/AT2-like cells that did not express *Tomato* and were sorted into ‘bulk’ samples were especially likely to be true negatives and were enriched for untransduced alveolar epithelial cells. We therefore removed these cells (which accounted for ~4% of all alveolar epithelial cells) before downstream analyses and considered the remaining AT1-, AT2- and AT1/AT2-like cells to be our cancer cell population.

### Differential gene expression analysis

Cell type-resolved differential gene expression analysis was performed using a pseudobulk approach to avoid potential issues stemming from pseudoreplication^77,78^. Pseudobulk aggregation was performed by summing unnormalized read counts per gene for each cell type in each sample using the decoupler function get_pseudobulk^79^. Pseudobulked samples corresponding to fewer than 10 cells or 1,000 counts were removed, and only cell types with at least two pseudobulked samples for each genotype-age category were analyzed.

Differential gene expression analysis was performed using DESeq2 (ref. ^80^) (v.1.42.0) with unnormalized pseudobulked counts as input. Count tables were filtered separately for each cell type to include only genes with at least ten counts across at least three samples. We performed two sets of analyses for each cell type: the first tested the effect of age on gene expression for each cell type separately within the sg*Inert* and sg*Pten* samples (Expression ~ Age); the second tested the effect of *Pten* inactivation on gene expression separately within the young and aged samples (Expression ~ Pten^−/−^). For all analyses we used the standard DESeq2 workflow with log fold change shrinkage and reported differentially expressed genes as those with a Benjamini–Hochberg-corrected Wald test *P* < 0.05.

### Signatures of normal (noncancer) aging and senescence

To assess the extent to which the transcriptional changes in our dataset aligned with pre-existing data on normal (noncancer) aging, we identified three pre-existing aging signatures: (1) genes upregulated with age in AT2 cells in a single-cell transcriptomic analysis of mouse lung (‘Angelidis’)^38^; (2) genes upregulated with age in AT2 cells in the Tabula Muris Senis (‘Tabula Muris’)^23^; and (3) genes that are upregulated with age in >80% of cell types in a pan-cell type re-analysis of the Tabula Muris Senis dataset (‘Global Aging’)^42^. In addition, we evaluated several pre-existing signatures of senescence and the senescence-associated secretory phenotype, including a gene set shown to identify senescence across tissues (‘SenMayo’)^46^, a lung epithelial cell-specific senescence signature (‘DePianto’)^45^, a gene set previously used to evaluate senescence across cancer types (‘Zhao’)^44^, as well as the Gene Ontology Biological Process and Reactome senescence genes sets from the Molecular Signatures Database^43^. We assessed agreement of these signatures with the transcriptional changes in our dataset through gene set enrichment analyses and per-cell gene set activation scoring (see below).

### Gene set enrichment analysis and gene set activation scoring

Gene set enrichment analysis (GSEA) was performed per cell type using GSEA v.4.3.2 with default parameters^81,82^. Genes were ranked by −log_10_(Wald *P* value) multiplied by the sign of the log fold change from DESeq2, and compared to gene sets corresponding to signatures of normal aging (see above). GSEApy (v. 1.1.1) was used to visualize gene set enrichments.

To complement our GSEA, which was based on the results of differential gene expression analysis using pseudobulked samples, we also performed per-cell scoring of aging gene sets. Specifically, we used the score_genes method implemented in scanpy, which quantifies the extent to which a gene set is active in each cell by subtracting the average expression of a reference set of genes randomly sampled across binned expression levels from the average expression of the gene set. This method was also used to infer cell cycle phase based on scoring of S and G2M phase genes using the scanpy function score_genes_cell_cycle^83^.

### Pathway activation analysis

PROGENy was used to estimate changes in signaling pathway activity with age and *Pten* inactivation based on changes in gene expression associated with these perturbations. This approach models signaling pathways as sets of target genes with interaction weights corresponding to the direction and extent of change in expression expected upon pathway activation^47^. For each perturbation, we used the decoupler Python package (v.1.5.0) to fit a multivariate linear model predicting DESeq2 log_2_ fold changes in gene expression based on PROGENy pathway-gene weights^79^. Reported pathway activity scores are the *t*-values for the model coefficients for each pathway.

### Statistics and reproducibility

The statistical tests used for each analysis are described in detail in the sections above. All analyses of barcode sequencing data were performed in Python (v.3.6.4) and visualizations of data were performed in Python (v.3.6.4) and R (v.4.3.2). Sample sizes were determined on the basis of our previous experience conducing similar experiments and, in the case of barcode sequencing experiments, on the basis of previously published power analyses^19^. The Investigators were not blinded to allocation during experiments and outcome assessment. No randomization was used in this study; data collection was blocked to ensure that experimental groups were roughly evenly represented across timepoints. No data were excluded from the analyses except where discussed in the ‘Removal of mice with aberrantly few barcodes’ and ‘Filtering of vectors with insufficient titer from analysis’ sections. Analyses of barcode sequencing data used nonparametric statistics; therefore, no assumptions about the distribution of data were made. Other metrics of tumorigenesis (for example lung weight, tumor burden and tumor number) were compared using Wilcoxon rank-sum tests. For analyzing immunohistochemical data, *t*-tests were used in these cases data distribution was assumed to be normal but this was not formally tested. Two-way ANOVAs were used to analyze interactions between age and tumor genotype, homogeneity of variance was confirmed by Levene’s test and normality was confirmed by the Shapiro–Wilk test.

### Reporting summary

Further information on research design is available in the Nature Portfolio Reporting Summary linked to this article.

## Supplementary information

Supplementary Information Supplementary Figs. 1–7.

Reporting Summary

Supplementary Table 1 sgRNA sequences and corresponding identifying sequences used in this study.

Supplementary Table 2 Results of differential gene expression analysis comparing young and old cancer cells from sg*Inert* samples.

## Source data

Source Data Fig. 1 Statistical source data.

Source Data Fig. 2 Statistical source data.

Source Data Fig. 3 Statistical source data.

Source Data Fig. 4 Statistical source data.

Source Data Extended Data Fig. 1 Statistical source data.

Source Data Extended Data Fig. 2 Statistical source data.

Source Data Extended Data Fig. 3 Statistical source data.

Source Data Extended Data Fig. 4 Statistical source data.

Source Data Extended Data Fig. 5 Statistical source data.

Source Data Extended Data Fig. 6 Statistical source data.

Source Data Extended Data Fig. 7 Statistical source data.

Source Data Extended Data Fig. 8 Statistical source data.

Supplementary Data 2 Statistical source data.

Source Data Extended Data Fig. 10 Statistical source data.

## Data Availability

All barcode sequencing datasets are available through the NCBI’s Sequence Read Archive Database under BioProject accession number PRJNA1261442. All scRNA-seq data are available through the Gene Expression Omnibus under the record GSE297023. Processed data plotted in figures are available as Source data.

## Code availability

The code used for data analysis in this study is available on GitHub (https://github.com/eshuldiner/Aging).
