# Supplementary material for: Aging represses oncogenic KRAS-driven lung tumorigenesis and alters tumor suppression
Source: Nat Aging. 2025 Nov 4;5(11):2263–78. doi: 10.1038/s43587-025-00986-z (PMC12616358; doi:10.1038/s43587-025-00986-z)
Supplement: Supplementary file 1 — Supplementary Figs. 1–7. [file 43587_2025_986_MOESM1_ESM.pdf]

nature aging

Supplementary information https://doi.org/10.1038/s43587-025-00986-z

Aging represses oncogenic KRAS-driven lung tumorigenesis and alters tumor suppression

In the format provided by the authors and unedited

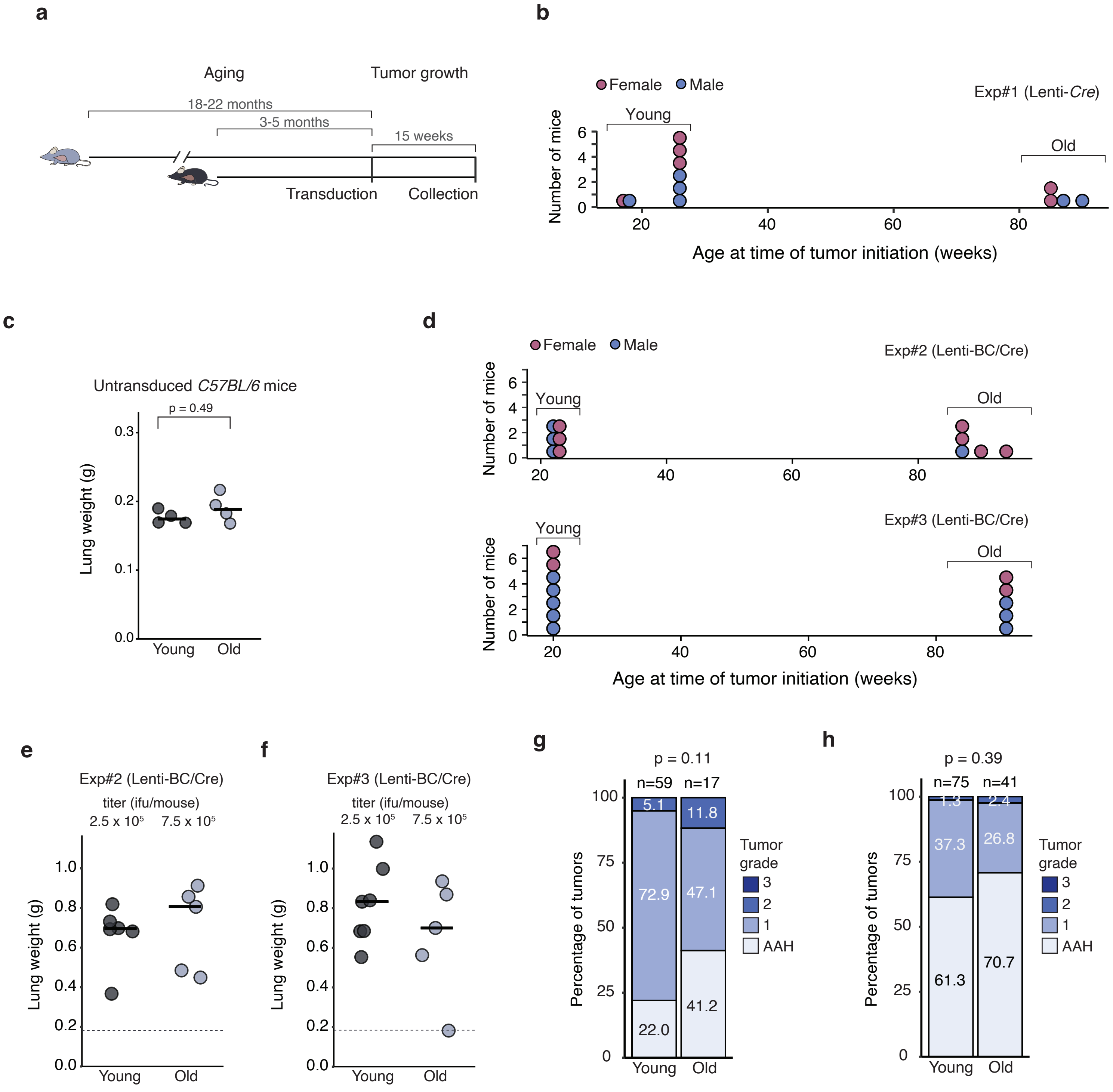

Supplementary Fig. 1. Additional data on generation of KRAS-driven tumors in young and old mice

a. Schematic of tumor initiation and collection in young and old mice. Mice in young and old cohorts were aged for indicated durations before simultaneous tumor initiation by intratracheal delivery of lentiviral vectors. Tumors were allowed to develop in both groups for 15 weeks before collection and analysis.

b. Ages of young (N=8) and old (N=4) mice transduced with Lenti-*Cre* in Exp #1 (**Fig. 1a**). Each dot is a mouse; color denotes sex.

c. Lung weights of untransduced young (N=4) and old (N=4) control mice matched to the ages of mice in Exp #1. Each dot is a mouse and the bars indicate the median values. P-value: two-sided Wilcoxon rank sum test.

d. Ages of young (N=6, 7) and old mice (N=5, 5) transduced with Lenti-*BC/Cre* in Exp #2 (top) and Exp #3 (bottom) (**Fig. 1f**). Each dot is a mouse; color denotes sex.

e,f. Lung weights of mice (N=6, 7 young and N=5, 5 old) transduced with Lenti-*BC/Cre* in Exp #2 (**e**) and Exp #3 (**f**). Each dot is a mouse and the bars indicate the median values. Median normal lung weight from (**c**) is indicated with dashed line.

g,h. Bar plots showing the percentage of tumors with the indicated grades in Exp #1 (**g**) and Exp #3 (**h**). The number of tumors graded is indicated above each bar; tumors were from N=4 mice per group. P-values: two-sided Fisher Exact test. AAH: atypical adenomatous hyperplasia.

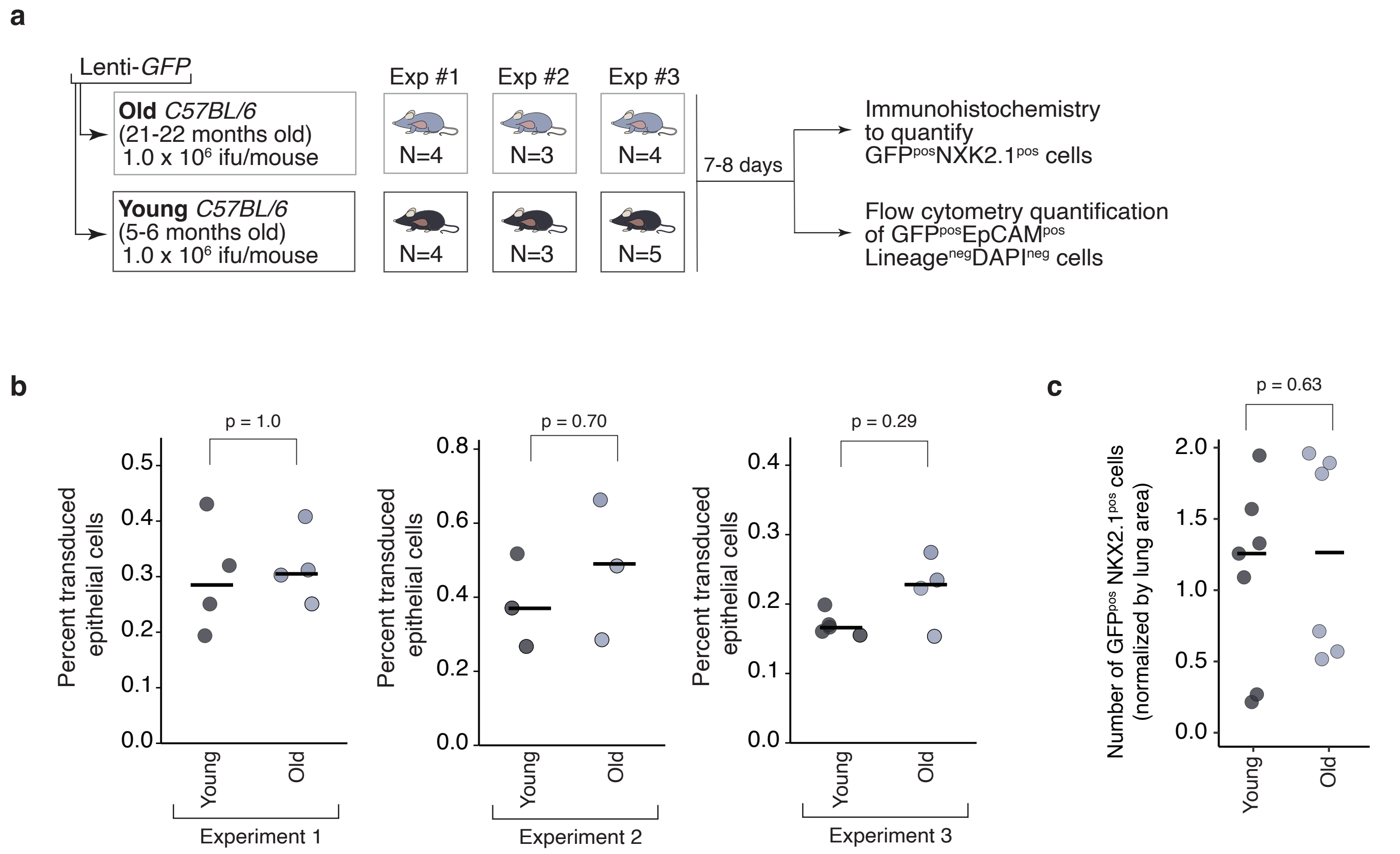

Supplementary Fig. 2. Lentiviral transduction efficiency of lung epithelial cells is not impacted by age

a. Transduction of young and old *C57BL/6* mice with Lenti-*GFP* to quantify lentiviral transduction efficiency. Lentiviral titer is indicated. Mice were analyzed 7-8 days after transduction. Lung lobes were either paraffin-embedded for immunohistochemical analysis or dissociated for analysis by flow cytometry. Lobes analyzed by immunohistochemistry were stained for GFP and lung epithelial cell lineage-defining transcription factor NKX2.1. Lobes analyzed by flow cytometry were stained for GFP, EpCAM and lineage markers (CD31, CD45, and F4/80) to identify epithelial cells, as well as DAPI to exclude dead cells. Age and number of mice are indicated.

b. Quantification of the percent of $EpCAM^{pos}Lineage^{neg}DAPI^{neg}$ cells that were GFP^{pos} (transduced) in young (N=4, 3, 5) and old (N=4, 3, 4) mice across three replicate experiments. Each dot represents a mouse and the bars indicate the median values. P values, two-sided Wilcoxon rank sum tests.

c. Quantification of GFP^{pos} and $NKX2.1^{pos}$ cells normalized by lung area in young (N=12) and old (N=11) mice. Each dot represents a mouse and the bars indicate median values. P value: two-sided Wilcoxon rank sum test.

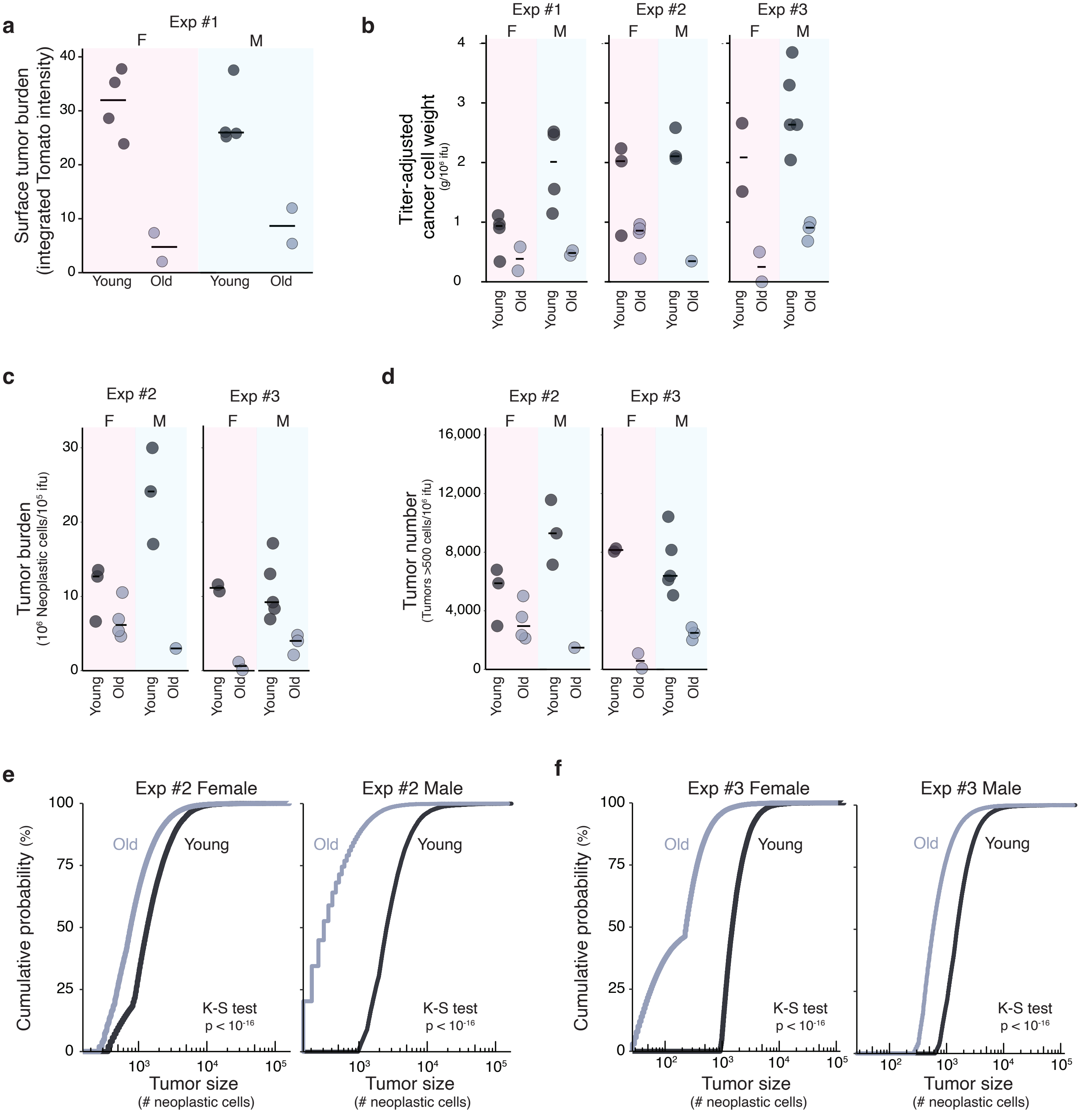

Supplementary Fig. 3. Aging represses KRAS-driven lung tumor initiation and growth in both males and females
a. Fluorescence-based quantification of tumor burden in young and old mice in Experiment #1 (transduced with Lenti-*Cre*) split by sex (N=4 female and 4 male young mice, N=2 female and 2 male old mice). Each dot is a mouse and the bars indicate median values.
b. Estimated cancer cell weights of young and old mice in Experiments #1-3 (transduced with Lenti-*Cre* and Lenti-*BC/Cre*), normalized to the viral titer delivered to each mouse and split by sex (N=4, 3, 2 female and 4, 3, 5 male young mice; N=2, 4, 2 female and 2, 1, 3 male old mice). Each dot is a mouse and the bars indicate median values.
c,d. Tumor burden (total number of neoplastic cells in clonal expansions > 500 cells quantified by Tuba-seq) (**c**), and number of tumors (clonal expansions >500 cells) (**d**) in young and old mice in Experiments #2 and #3 transduced with with Lenti-*BC/Cre* normalized to the viral titer delivered to each mouse and split by sex (N=3, 2 female and 3, 5 male young mice; N=4, 2 female and 1, 3 male old mice). Each dot is a mouse and the bars indicate median values.
e,f. Empirical cumulative distribution functions of tumor sizes in young and old mice from Experiment #2 (**e**) and Experiment #3 (**f**) split by sex (N=3, 2 female and 3, 5 male young mice; N=4, 2 female and 1, 3 male old mice). To account for the 3-fold higher titer delivered to the old mice, this comparison includes the 10,000 largest tumors in each young sample and the 30,000 largest tumors from each old sample. Tumors in old mice are smaller than tumors in young mice irrespective of sex. K-S test: two-sided asymptotic Kolmogorov-Smirnov test.

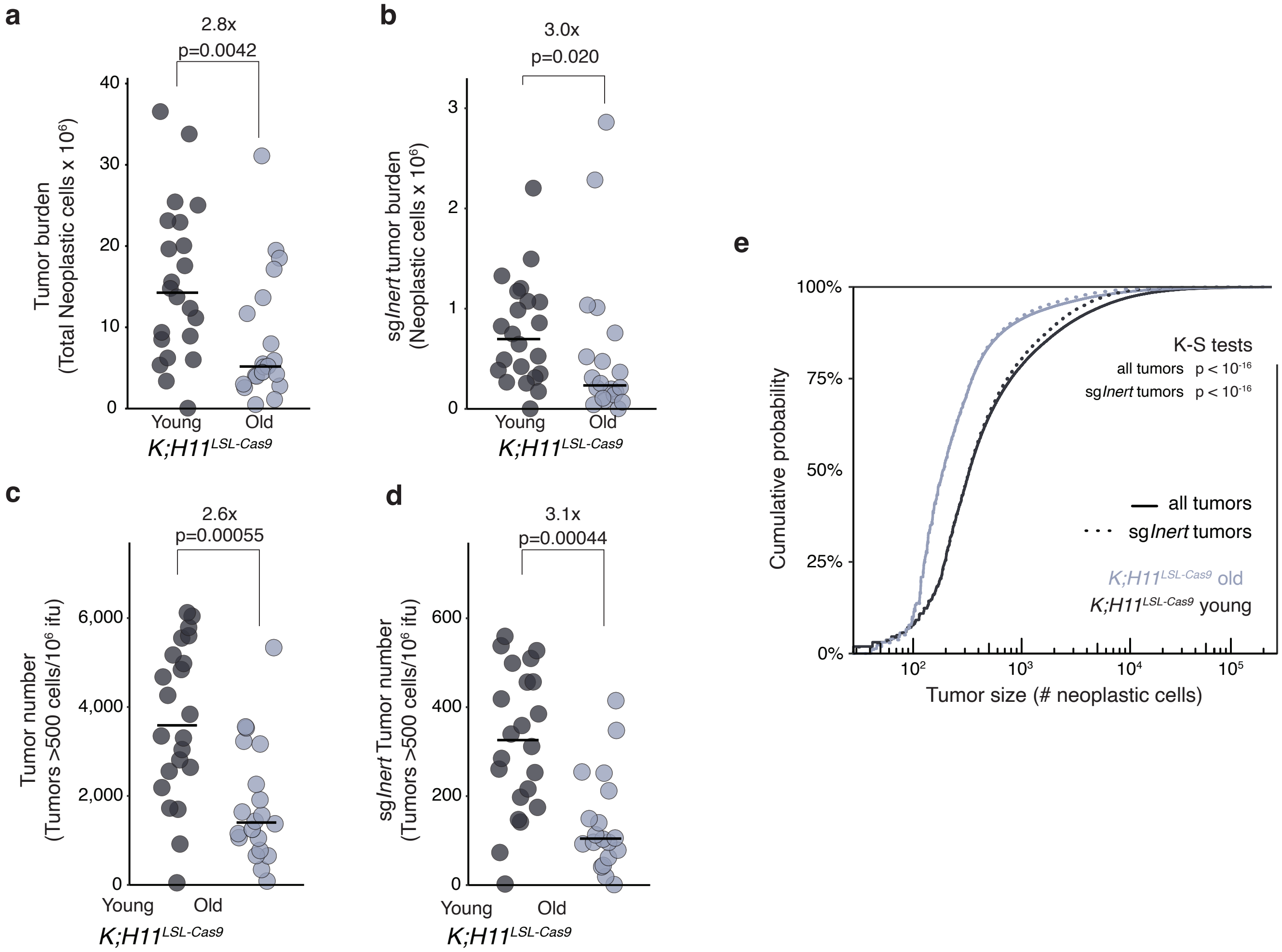

Supplementary Fig. 4. Pooled screen of tumor suppressor function recapitulates suppressive effect of aging on KRAS-driven lung tumorigenesis

a-b. Tumor burden (total number of neoplastic cells in clonal expansions > 500 cells) across all Lenti-sg*RNA/Cre* vectors (**a**) and across only sg*Inert* vectors (**b**) in young and old *K;H11*$^{LSL\text{-}Cas9}$ mice transduced with the Lenti-sg*RNA*^{Aging}*/Cre* pool.

c-d. Number of tumors (clonal expansions > 500 cells) across all Lenti-sg*RNA/Cre* vectors (**c**) and across only sg*Inert* vectors (**d**) in young and old *K;H11*$^{LSL\text{-}Cas9}$ mice transduced with the Lenti-sg*RNA*^{Aging}*/Cre* pool.For **a**-**d**: Each dot is a mouse. Bars indicate median value within each age group. P-values were calculated using two-sided Wilcoxon rank sum tests.

e. Cumulative distribution functions of tumor size across all Lenti-sg*RNA/Cre* vectors ("all tumors") and across Lenti-sg*Inert/Cre* vectors ("sg*Inert* tumors") in young and old *K;H11*$^{LSL\text{-}Cas9}$ mice transduced with the Lenti-sg*RNA*^{Aging}*/Cre* pool. Distribution for all tumors was constructed by sampling the 10,000 largest tumors per young and old mouse (irrespective of Lenti-sg*RNA/Cre* vector); distribution for *sgInert* tumors was constructed using the subset of the 10,000 largest tumors per mouse that contained Lenti-sg*Inert/Cre* vectors. K-S tests: two-sided asymptotic Kolmogorov-Smirnov tests comparing the overall distribution and the distribution of sg*Inert* tumor sizes between young and old mice.

All panels: N = 22 young and 20 old *K;H11*$^{LSL\text{-}Cas9}$ mice.

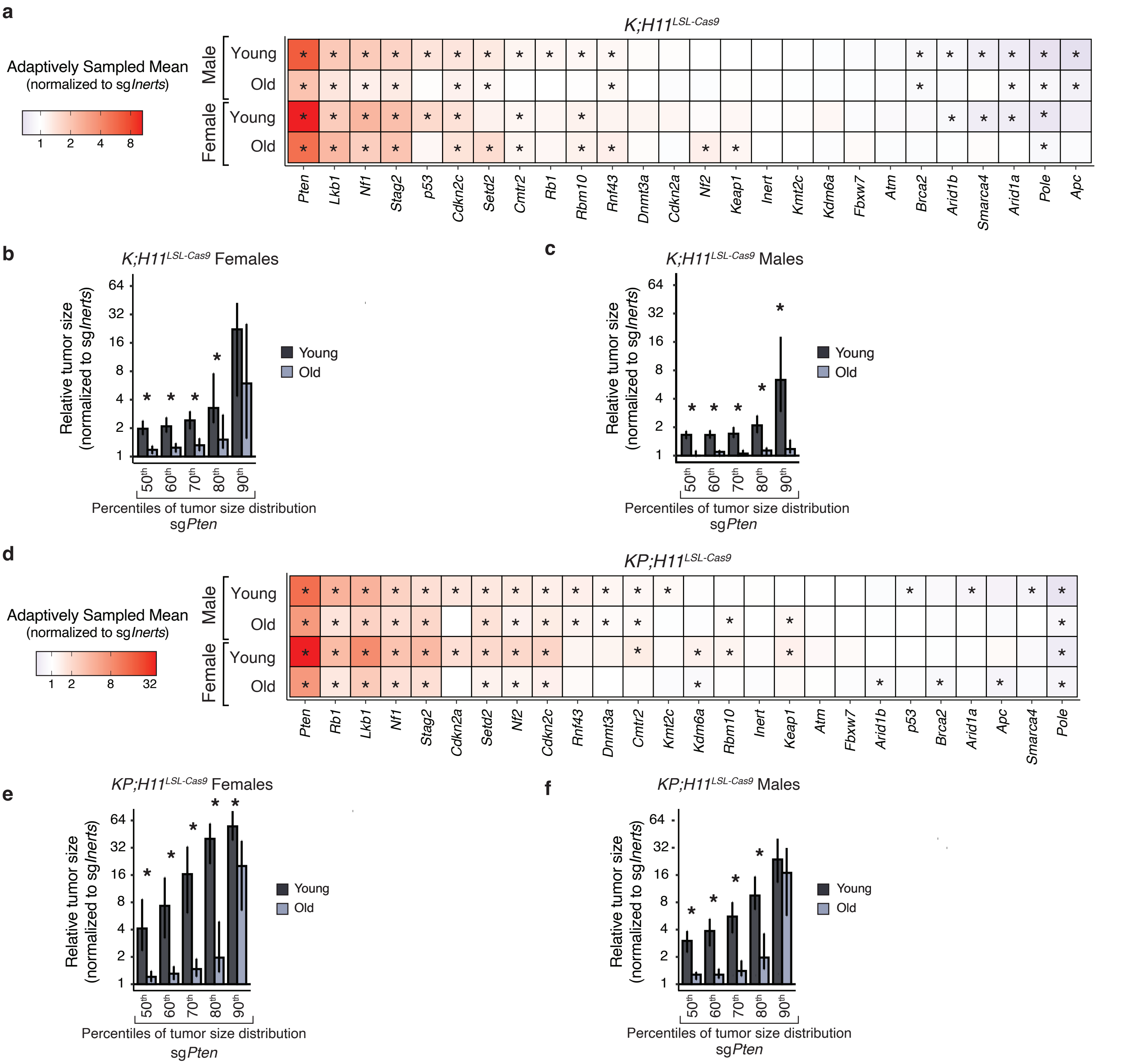

Supplementary Fig. 5. Differential effects of tumor suppressor gene inactivation with age in males and females

a. Adaptively sampled mean (ASM) tumor sizes normalized to sg*Inert* for all tumor genotypes in male (top) and female (bottom) young and old $K;H11^{LSL\text{-}Cas9}$ mice transduced with the Lenti-sgRNA^{Aging}/*Cre* pool. Genes are ordered by ASM in young males. Stars denote that gene significantly impacts ASM relative to sg*Inerts* (two-sided FDR-adjusted p-value < 0.05).

b, c. Adaptively sampled sizes of tumors initiated with sg*Pten* vectors in young and old $K;H11^{LSL\text{-}Cas9}$ female (**b**) and male (**c**) mice at indicated percentiles of the tumor size distribution. Each statistic is normalized to tumor size at the corresponding percentile of the sg*Inert* distribution. Stars denote a statistically significant difference between young and old (two-sided FDR-adjusted p-value < 0.05). P= 0.046, 0.25 for 80th, 90th percentiles for $K;H11^{LSL\text{-}Cas9}$ females; $P<10^{-4}$ for all other comparisons.

d. ASM tumor sizes normalized to sg*Inert* for all tumor genotypes in male (top) and female (bottom) young and old $KP;H11^{LSL\text{-}Cas9}$ mice transduced with Lenti-sgRNA^{Aging}/*Cre*. Genes are ordered by ASM in young males. Stars denote that gene significantly impacts ASM relative to sg*Inerts* (two-sided FDR-adjusted p-value < 0.05).

e,f. Adaptively sampled sizes of tumors initiated with sg*Pten* vectors in young and old $KP;H11^{LSL\text{-}Cas9}$ female (**e**) and male (**f**) mice at indicated percentiles of the tumor size distribution. Each statistic is normalized to tumor size at the corresponding percentile of the sg*Inert* distribution. Stars denote a statistically significant difference between young and old (two-sided FDR-adjusted p-value < 0.05). P=0.0048 for 90th percentile for $KP;H11^{LSL\text{-}Cas9}$ females, P=0.00025, 0.45 for 80th and 90th percentiles for $KP;H11^{LSL\text{-}Cas9}$ males; $P<10^{-4}$ for all other comparisons.

a-c: N=9 female and 13 male young $K;H11^{LSL\text{-}Cas9}$ mice; N=11 female and 9 male old $K;H11^{LSL\text{-}Cas9}$ mice.

d-f: N=9 female and 7 male young $KP;H11^{LSL\text{-}Cas9}$ mice; N=12 female and 13 male old $KP;H11^{LSL\text{-}Cas9}$ mice.

All panels: Errors bars indicate 95% confidence intervals around the point estimate of the test statistic (center of bars). P-values and confidence intervals were calculated using nested bootstrap resampling.

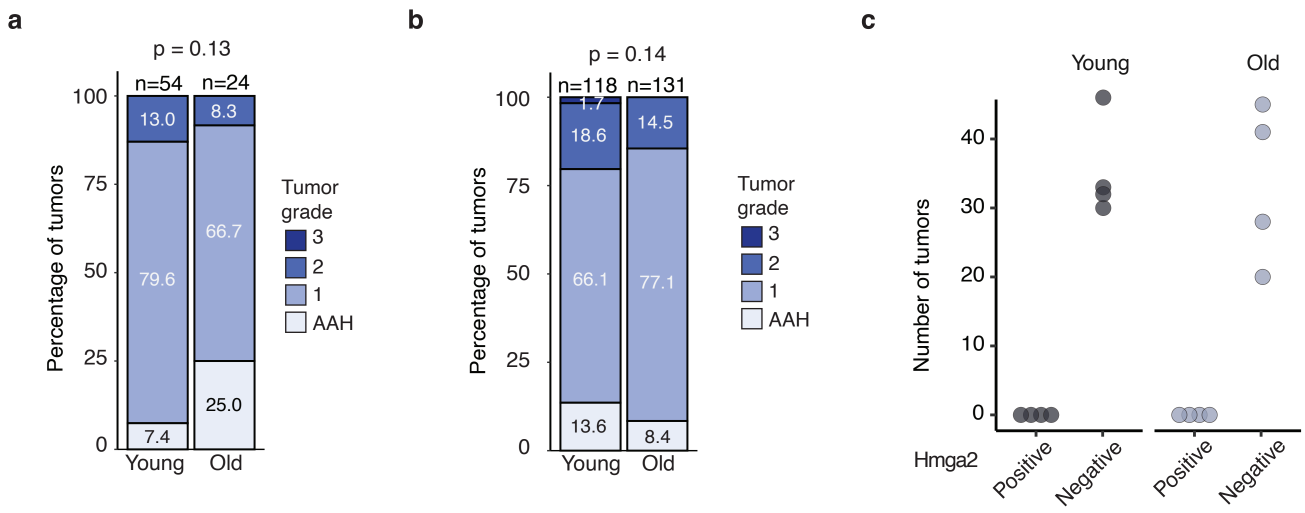

Supplementary Fig. 6. Age does not impact the grade of PTEN-deficient oncogenic KRAS-driven lung tumors
a,**b**. Bar plots showing the percentage of tumors with the indicated grades in mice transduced with Lenti-sg*Pten/Cre* (**a**) and Lenti-sg*Pten-Pool/Cre* (**b**). Number of tumors graded is indicated above each bar; tumors were from N=4 mice per group. P-value: two-sided Fisher Exact test. Note that these mice were analyzed at the same time point and graded alongside (and can thus be directly compared to) the data in **Supplementary Fig. 1g**,**h**.
c. Numbers of PTEN-deficient tumors staining positive or negative for HMGA2 (N=4 mice per group).

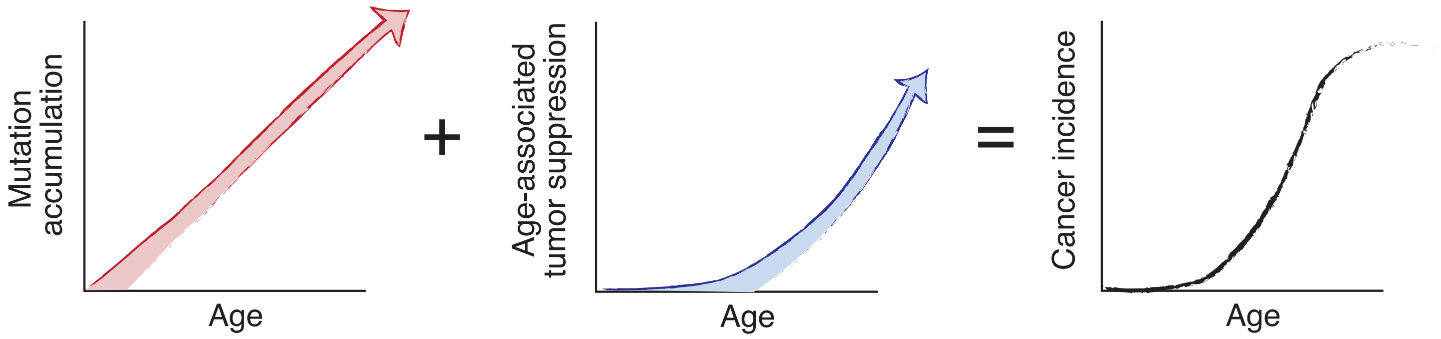

Supplementary Fig 7. Model for integration of the effects of mutation accumulation and age-associated tumor supression to produce non-monotonic pattern of cancer incidence with age
Mutations (red arrow) accumulate with age, driving increasing cancer incidence throughout much of the lifespan. In old age, the tumor-repressive effects of aging (blue arrow) overtake the pro-tumorigenic effects of mutation accumulation, resulting in deceleration and eventual decrease in cancer incidence.
