# Supplementary material for: Aging represses oncogenic KRAS-driven lung tumorigenesis and alters tumor suppression
Source: Nat Aging. 2025 Nov 4;5(11):2263–78. doi: 10.1038/s43587-025-00986-z (PMC12616358; doi:10.1038/s43587-025-00986-z)
Supplement: Supplementary file 2 — Reporting Summary [file 43587_2025_986_MOESM2_ESM.pdf]

nature portfolio

Corresponding author(s): Dmitri A. Petrov
Monte M. Winslow

Last updated by author(s): August 3, 2025

Reporting Summary

Nature Portfolio wishes to improve the reproducibility of the work that we publish. This form provides structure for consistency and transparency in reporting. For further information on Nature Portfolio policies, see our Editorial Policies and the Editorial Policy Checklist.

Statistics

For all statistical analyses, confirm that the following items are present in the figure legend, table legend, main text, or Methods section.

n/a	Confirmed	
☐	☒	The exact sample size (n) for each experimental group/condition, given as a discrete number and unit of measurement
☐	☒	A statement on whether measurements were taken from distinct samples or whether the same sample was measured repeatedly
☐	☒	The statistical test(s) used AND whether they are one- or two-sided<br>*Only common tests should be described solely by name; describe more complex techniques in the Methods section.*
☒	☐	A description of all covariates tested
☐	☒	A description of any assumptions or corrections, such as tests of normality and adjustment for multiple comparisons
☐	☒	A full description of the statistical parameters including central tendency (e.g. means) or other basic estimates (e.g. regression coefficient) AND variation (e.g. standard deviation) or associated estimates of uncertainty (e.g. confidence intervals)
☐	☒	For null hypothesis testing, the test statistic (e.g. F, t, r) with confidence intervals, effect sizes, degrees of freedom and P value noted<br>*Give P values as exact values whenever suitable.*
☒	☐	For Bayesian analysis, information on the choice of priors and Markov chain Monte Carlo settings
☒	☐	For hierarchical and complex designs, identification of the appropriate level for tests and full reporting of outcomes
☐	☒	Estimates of effect sizes (e.g. Cohen's d, Pearson's r), indicating how they were calculated

Our web collection on statistics for biologists contains articles on many of the points above.

Software and code

Policy information about availability of computer code

Data collection	No software was used to collect data.
Data analysis	Python 3.6.4 was used for all analyses of barcode sequencing data. Python 3.6.24 and R 4.3.2 were used for data visualization. Decoupler (version 1.5.0), DESeq2 (version 1.42.0), SCINA (version 1.2.0), scrublet (version 0.2.3), GSEA (version 4.3.2), GSEApy (version 1.1.1), scanpy (version 1.9.8), and split-pipe (version 1.1.1) were used in analyzing the scRNA-sequencing data. ImageJ (version 1.53) was used for analysis of fluorescence images. QuPath (version 0.5.1) was used for analysis of CD45, B220, CD4 and CD8 immunohistochemistry. Code used in the analysis of barcode sequencing data and single-cell RNA-sequencing analysis is publicly available on GitHub (https://github.com/eshuldiner/Aging).

For manuscripts utilizing custom algorithms or software that are central to the research but not yet described in published literature, software must be made available to editors and reviewers. We strongly encourage code deposition in a community repository (e.g. GitHub). See the Nature Portfolio guidelines for submitting code & software for further information.

Data

Policy information about availability of data

All manuscripts must include a data availability statement. This statement should provide the following information, where applicable:

- Accession codes, unique identifiers, or web links for publicly available datasets
- A description of any restrictions on data availability
- For clinical datasets or third party data, please ensure that the statement adheres to our policy

All barcode sequencing datasets are available through the NCBI's Sequence Read Archive Database under the BioProject accession number PRJNA1261442. All single-cell RNA sequencing data are available through the Gene Expression Omnibus under the record GSE297023. Processed data plotted in figures are available as Source Data.

Research involving human participants, their data, or biological material

Policy information about studies with human participants or human data. See also policy information about sex, gender (identity/presentation), and sexual orientation and race, ethnicity and racism.

Reporting on sex and gender

Use the terms sex (biological attribute) and gender (shaped by social and cultural circumstances) carefully in order to avoid confusing both terms. Indicate if findings apply to only one sex or gender; describe whether sex and gender were considered in study design; whether sex and/or gender was determined based on self-reporting or assigned and methods used.
Provide in the source data disaggregated sex and gender data, where this information has been collected, and if consent has been obtained for sharing of individual-level data; provide overall numbers in this Reporting Summary. Please state if this information has not been collected.
Report sex- and gender-based analyses where performed, justify reasons for lack of sex- and gender-based analysis.

Reporting on race, ethnicity, or other socially relevant groupings

Please specify the socially constructed or socially relevant categorization variable(s) used in your manuscript and explain why they were used. Please note that such variables should not be used as proxies for other socially constructed/relevant variables (for example, race or ethnicity should not be used as a proxy for socioeconomic status).
Provide clear definitions of the relevant terms used, how they were provided (by the participants/respondents, the researchers, or third parties), and the method(s) used to classify people into the different categories (e.g. self-report, census or administrative data, social media data, etc.)
Please provide details about how you controlled for confounding variables in your analyses.

Population characteristics

Describe the covariate-relevant population characteristics of the human research participants (e.g. age, genotypic information, past and current diagnosis and treatment categories). If you filled out the behavioural & social sciences study design questions and have nothing to add here, write "See above."

Recruitment

Describe how participants were recruited. Outline any potential self-selection bias or other biases that may be present and how these are likely to impact results.

Ethics oversight

Identify the organization(s) that approved the study protocol.

Note that full information on the approval of the study protocol must also be provided in the manuscript.

Field-specific reporting

Please select the one below that is the best fit for your research. If you are not sure, read the appropriate sections before making your selection.

☒ Life sciences ☐ Behavioural & social sciences ☐ Ecological, evolutionary & environmental sciences

For a reference copy of the document with all sections, see nature.com/documents/nr-reporting-summary-flat.pdf

Life sciences study design

All studies must disclose on these points even when the disclosure is negative.

Sample size

Sample sizes were determined based on our previous experience (e.g. Rogers, McFarland, Winters et al. Nature Genetics; Cai, Chew, Li et al. Cancer Discovery, 2021; Tang and Shuldiner et al. Nature Cell Biology 2023) and power calculations (Cai et al. Cancer Discovery 2021).

Data exclusions

As discussed in the Methods, 3 mice were excluded from analysis of barcode sequencing data due to extremely poor sequencing depth and 4 mice were excluded due to extremely low barcode numbers. Reasoning behind these exclusions is explained in the Methods. These criteria were not established prior to the study; however, we based these exclusions on the overall distributions of sequencing depth and barcode number blinded to mouse age (our primary variable of interest), so we do not believe this introduced bias. In addition, a small number of vectors with titers roughly an order of magnitude lower than the median titer were excluded from the analysis. This criterion was not established prior to the study, however, the relative titer of vectors were assessed in mice lacking Cas9 in which the vectors were functionally equivalent, therefore we do not anticipate that this introduced bias.

Replication

We performed extensive biological replication (at least 4 mice per cohort, and multiple replicate experiments to confirm key findings). The

Replication	age-dependent effect of Pten inactivation was confirmed across 3 sgRNAs and four separate paired cohorts of mice. Our findings are consistent across replicates.
Randomization	Formal sample randomization was not performed in this study. However, we allocated mice to ensure that all experimental cohorts were sex balanced, thereby controlling for this covariate.
Blinding	For reasons of practicality, experiments were not blinded during mouse handling and analyses that required directly handling samples. Downstream analyses of barcode sequencing and single cell RNA-sequencing data were performed using pipelines that were applied identically to all samples; thus we do not anticipate these steps introduced bias. Likewise, an identical set of steps was applied to each sample in analyzing fluorescence and histological data (and the identity of samples was blinded in determining these steps); thus we do not anticipate bias in these analyses.

Reporting for specific materials, systems and methods

We require information from authors about some types of materials, experimental systems and methods used in many studies. Here, indicate whether each material, system or method listed is relevant to your study. If you are not sure if a list item applies to your research, read the appropriate section before selecting a response.

Materials & experimental systems

n/a	Involved in the study	
☐	☒	Antibodies
☒	☐	Eukaryotic cell lines
☒	☐	Palaeontology and archaeology
☐	☒	Animals and other organisms
☒	☐	Clinical data
☒	☐	Dual use research of concern
☒	☐	Plants

Methods

n/a	Involved in the study	
☒	☐	ChIP-seq
☒	☐	Flow cytometry
☒	☐	MRI-based neuroimaging

Antibodies

Antibodies used	Antibodies against CD45 (BioLegend, 103112 1:800 dilution for FACS and Cell Signaling, 70257S clone D3F8Q 1:200 dilution for immunohistochemistry), CD31 (BioLegend, 102410; 1:800 dilution), F4/80 (BioLegend, 123116; 1:800 dilution), Ter119 (BioLegend, 116212; 1:800 dilution), B220 (BD Biosciences, 550286 clone RA3-6B2; 1:1500 dilution), CD4 (Cell Signaling, 25229S clone D7D2Z; 1:200 dilution), CD8 (Cell Signaling, 98941S clone D4W2Z; 1:200 dilution), NKX2.1 (Abcam, ab76013; 1:250 dilution), HMGA2 (Biocheck, 59170AP; 1:500 dilution), and GFP (Cell Signaling Technology, mAb #2956; 1:100 dilution) were used.
Validation	Use of these antibodies to stain primary mouse tumor cells has been validated in previous projects from our lab (e.g. Murray et al. 2019 Cancer Discovery, Yousefi et al. 2022 Cancer Research).

Animals and other research organisms

Policy information about studies involving animals; ARRIVE guidelines recommended for reporting animal research, and Sex and Gender in Research

Laboratory animals	KrasLSL-G12D/+ (RRID:IMSR_JAX:008179), R26LSL-tdTomato (RRID:IMSR_JAX:007909),H11LSL-Cas9 (RRID:IMSR_JAX:027632), and p53flox/flox (RRID:IMSR_JAX008462) mice have been previously described. All mice were on a C57BL/6 background. The sex of animals was balanced in each cohort in each experiment. Ages of mice ranged from 2-8 months for young mice and 17-24 months for old mice at the time of tumor initiation; detailed ages are provided in the manuscript.
Wild animals	The study did not involve wild animals.
Reporting on sex	Sex was considered in study design by ensuring that all cohorts were sex-balanced. To assess the impact of sex on our findings we repeated analyses separately within males and females. All key findings of this study applied to both sexes. Specifically, tumor burden, number and size were reduced with age in both males and females (demonstrated in Supplementary Fig. 3). Likewise, the effects of tumor suppressor inactivation were weakened with age with Pten most strongly affected in both males and females (demonstrated in Supplementary Fig. 7).
Field-collected samples	The study did not involve samples collected in the field.
Ethics oversight	The use of mice for this study was approved by the Institutional Animal Care and Use Committee at Stanford University, protocol number 26696.

Note that full information on the approval of the study protocol must also be provided in the manuscript.

Plants

Seed stocks

Report on the source of all seed stocks or other plant material used. If applicable, state the seed stock centre and catalogue number. If plant specimens were collected from the field, describe the collection location, date and sampling procedures.

Novel plant genotypes

Describe the methods by which all novel plant genotypes were produced. This includes those generated by transgenic approaches, gene editing, chemical/radiation-based mutagenesis and hybridization. For transgenic lines, describe the transformation method, the number of independent lines analyzed and the generation upon which experiments were performed. For gene-edited lines, describe the editor used, the endogenous sequence targeted for editing, the targeting guide RNA sequence (if applicable) and how the editor was applied.

Authentication

Describe any authentication procedures for each seed stock used or novel genotype generated. Describe any experiments used to assess the effect of a mutation and, where applicable, how potential secondary effects (e.g. second site T-DNA insertions, mosiacism, off-target gene editing) were examined.
